# A Leveraged Signal-to-Noise Ratio (LSTNR) Method to Extract Differentially Expressed Genes and Multivariate Patterns of Expression From Noisy and Low-Replication RNAseq Data

**DOI:** 10.3389/fgene.2018.00176

**Published:** 2018-05-16

**Authors:** Oswaldo A. Lozoya, Janine H. Santos, Richard P. Woychik

**Affiliations:** Genome Integrity and Structural Biology Laboratory, National Institute of Environmental Health Sciences, National Institutes of Health, Durham, NC, United States

**Keywords:** DEG, RNAseq, LSTNR, noise, expression patterns, biomarker discovery

## Abstract

To life scientists, one important feature offered by RNAseq, a next-generation sequencing tool used to estimate changes in gene expression levels, lies in its unprecedented resolution. It can score countable differences in transcript numbers among thousands of genes and between experimental groups, all at once. However, its high cost limits experimental designs to very small sample sizes, usually *N* = 3, which often results in statistically underpowered analysis and poor reproducibility. All these issues are compounded by the presence of experimental noise, which is harder to distinguish from instrumental error when sample sizes are limiting (e.g., small-budget pilot tests), experimental populations exhibit biologically heterogeneous or diffuse expression phenotypes (e.g., patient samples), or when discriminating among transcriptional signatures of closely related experimental conditions (e.g., toxicological modes of action, or MOAs). Here, we present a leveraged signal-to-noise ratio (LSTNR) thresholding method, founded on generalized linear modeling (GLM) of aligned read detection limits to extract differentially expressed genes (DEGs) from noisy low-replication RNAseq data. The LSTNR method uses an agnostic independent filtering strategy to define the dynamic range of detected aggregate read counts per gene, and assigns statistical weights that prioritize genes with better sequencing resolution in differential expression analyses. To assess its performance, we implemented the LSTNR method to analyze three separate datasets: first, using a systematically noisy *in silico* dataset, we demonstrated that LSTNR can extract pre-designed patterns of expression and discriminate between “noise” and “true” differentially expressed pseudogenes at a 100% success rate; then, we illustrated how the LSTNR method can assign patient-derived breast cancer specimens correctly to one out of their four reported molecular subtypes (luminal A, luminal B, Her2-enriched and basal-like); and last, we showed the ability to retrieve five different modes of action (MOA) elicited in livers of rats exposed to three toxicants under three nutritional routes by using the LSTNR method. By combining differential measurements with resolving power to detect DEGs, the LSTNR method offers an alternative approach to interrogate noisy and low-replication RNAseq datasets, which handles multiple biological conditions at once, and defines benchmarks to validate RNAseq experiments with standard benchtop assays.

## Introduction

At their core, RNAseq and qPCR are two flavors of the same principle: by quantifying how many copies of different molecular templates are accumulated after a discrete number of duplication rounds, it is possible to estimate their abundance in the original sample as long as the amplification has occurred in exponential fashion (Livak and Schmittgen, 2001; Pfaffl, 2001). Viewed under this light, the realization that RNAseq data analysis offers the same challenges qPCR faces is somewhat expectable; nevertheless, discriminating good RNAseq data from bad is all but impossible if following the traditional in-depth inspection of qPCR data quality.

For starters, RNAseq can collect information from thousands of genes in a massively parallel fashion within a single experiment; given the scale of generated data, this means inspection of differential expression levels from each individual gene one at a time is impractical. Also, the discrete form of raw RNAseq output, i.e., countable reads, is of a different nature and statistical behavior than the output of other techniques, such as qPCR and hybridization microarrays, in the form of a digitized continuous-valued signal mass (Roy et al., 2011). Finally, in RNAseq experiments the expected variation in total number of representative reads for each detected transcript within a sample depends, among other factors, on each transcript's size, abundance, and GC-composition. Efforts to control the effects of such sequencing biases during library assembly have been posited early on during the development of RNAseq technologies (Auer and Doerge, 2010; Bullard et al., 2010); at the same time, development of various programmatic strategies to adjust against those biases during statistical analyses has continued ever since (Hansen et al., 2010; Aird et al., 2011; Risso et al., 2011; Benjamini and Speed, 2012; Wu et al., 2013; Law et al., 2014; Finotello and Di Camillo, 2015).

In many pipelines for RNAseq analysis, read outputs are transformed to a normalized measurement of relative expression between two samples, such as fold-change differences. However, performing read normalization can be problematic because it “divides out” the net output of detected reads. Without that information, it is impossible to determine whether observed experimental variation is consistent with the detection capacity of sequencing hardware or not. As a result, the ability to discern between a real signal and instrumental noise is lost. These issues are magnified in experiments with low replicate numbers—a common limitation that RNAseq users face due to costs of these technologies—or when specimens under inspection show highly heterogeneous transcriptional profiles within statistical groups (Hansen et al., 2011; Oberg et al., 2012; Robles et al., 2012). To this day, financial constraints to acquire and maintain RNAseq instrumentation, combined with complex structure of output data, remain the primary obstacles preventing RNAseq technologies to join clinical diagnostic settings when characterizing transcriptional profiles of patient-derived specimens (Nazarov et al., 2017).

Still, RNAseq remains the most powerful technique to assay gene expression genome-wide. The accuracy in gene expression measurements afforded by RNAseq relies on the volume and diversity of aggregated cDNA fragments, each distinguished from all others by their nucleotide sequences (Cloonan et al., 2008; Mortazavi et al., 2008; Oshlack et al., 2010); conversely, errors in RNAseq measurements are usually ascribed to nucleotide-integration errors during PCR amplification (Finotello and Di Camillo, 2015) because it is assumed that the exquisite sensitivity of detection hardware in next-generation sequencers guarantees accuracy. However, this assumption is not entirely correct: digital detection systems will generate output data from faithfully replicated sequences, trace contaminant templates and misconstrued sequences all the same depending on the quality of base-calling. This means that distinguishing detection noise that stems from PCR bias, instrumental error, contamination or any of them combined is not only difficult, but perhaps irrelevant—all of them happen, and all of them distort gene expression measurements the worst when calculated from low read counts. In other words, while base-calling in RNAseq depends on how precisely nucleotides are detected, measuring expression differences is a resolution problem.

Challenges to resolving expression differences by RNAseq are complicated further when the number of experimental samples is small and rates of detection for differentially expressed genes (DEGs) is poor. In those scenarios, the traditional route is to increase the sequencing depth for each sample with additional rounds of sequencing. Adding sequencing rounds surely increases the number of aligned reads per gene (or coverage); however, no matter how low the rate of misconstrued and contaminant reads that contribute to the total read output may be, they will amount to higher numbers of “phantom reads” undistinguishable from sequencing noise as more sequencing rounds pile up (Tarazona et al., 2011; Li and Tibshirani, 2013). This means that, without a threshold to discriminate the rate of contaminant templates that randomly align to a reference genome, the assumption that aligned reads measure the different cDNA copies in a sample's RNA pool is as “true” as assuming that they represent sequencing artifacts. Determining such a threshold of expectable detected artifacts from RNAseq data calls for a statistical treatment of raw sequencing output, one that deems collected reads as a combination of faithful and artefactual sequences that align to a reference genome.

Here, we present a leveraged signal-to-noise ratio (LSTNR) thresholding method, founded on generalized linear modeling (GLM) of aligned read detection limits to extract DEGs from count-based and noisy low-replication RNAseq experiments. The LSTNR method uses an agnostic independent filtering strategy to define the dynamic range of detected aggregate read counts per gene that can be explained by the variation in sequencing coverage across genes and experimental replicates. This approach not only determines a minimum read count density that true expressed genes must accrue for reliable detection, but also qualifies genes as more or less reliable for differential expression measurements based on how distant they are from the reliable detection minimum. By taking into account that expression measurements based on read counts have different levels of resolution for different genes, the LSTNR method relies on one fundamental difference between the nature of scale-dependent dispersion in sequencing output (uniquely aligned reads) and the behavior of differential expression estimates *secondary to the original output* (log-transformed relative fold changes between genes or treatment groups): that RNAseq-based estimates of gene expression differences between groups are as robust as the sequencing depth that underlie them.

## Materials and methods

### Implementation of LSTNR method

**Figure 1:**
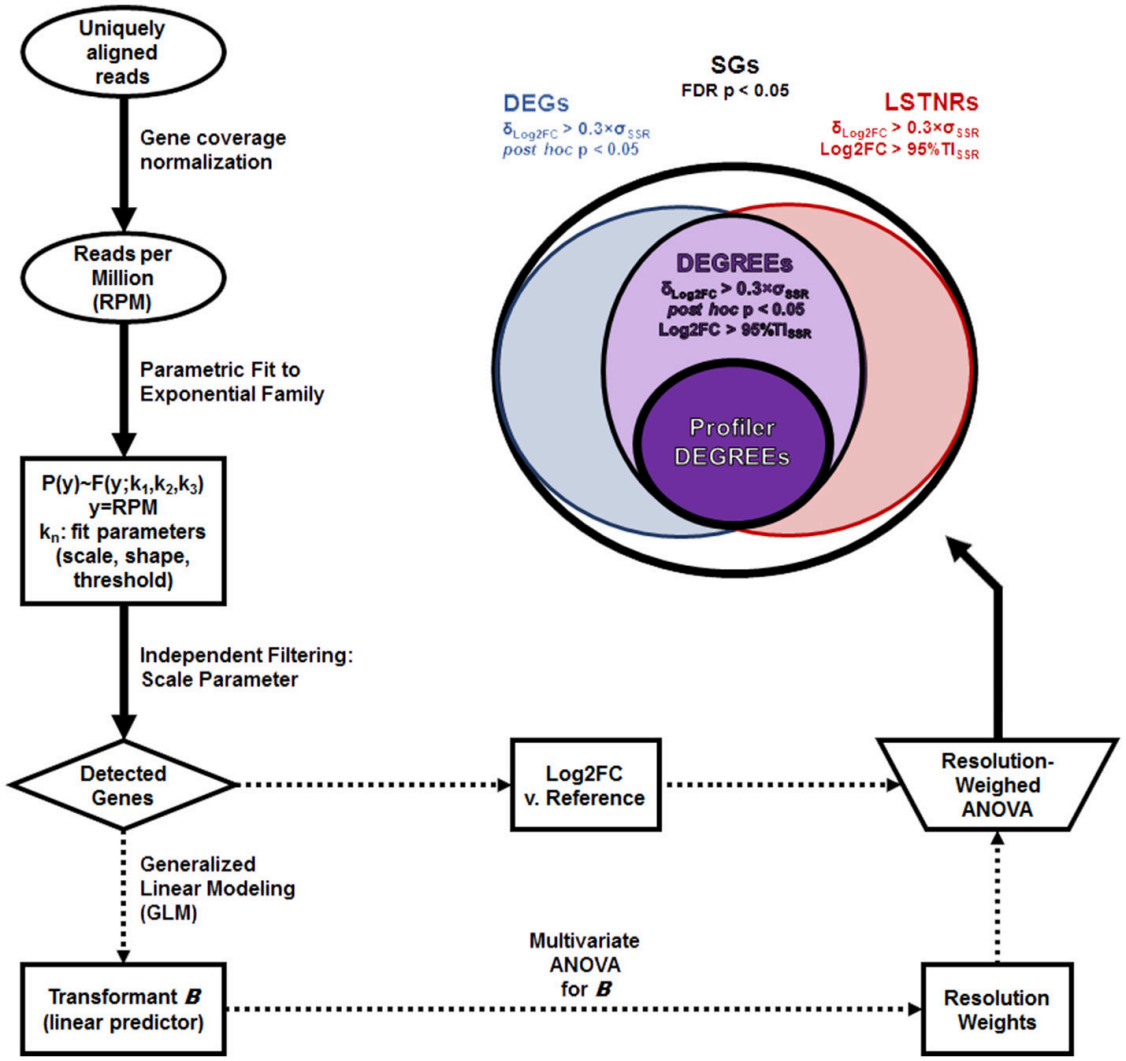
LSTNR method workflow. The schematic depicts the main steps involved in detection of statistically significant genes (SGs) starting from uniquely aligned reads for each gene in individual samples: coverage normalization as reads per million total sequenced reads (RPM); parametric distribution fitting; independent filtering based on fit parameters; generalized linear modeling (GLM), leading to gene resolution weights; log-fold expression measurements (Log2FC) vs. a fixed reference; and two-way resolution-weighed ANOVA of Log2FC. Top right: stratification criteria for SGs and their respective nomenclature: differentially expressed genes (DEGs), leveraged signal-to-noise-ratio genes (LSTNRs), and DEGs with reproducible expectation estimates (DEGREEs). The subset of Profiler DEGREEs corresponds to DEGREEs with a mean retrospective statistical power ≪π≫ > 90% which, when ranked by within-gene observed effect sizes (Δ_Log2FC_) first and by their 95% lower confidence limit of retrospective statistical power (π_low_) next, show monotonically decreasing Δ_Log2FC_ values. Profiler DEGREEs can then be used for benchtop validation or for additional statistical analysis to obtain a reductive set of prospective biomarkers through a variety of machine-learning analytics (e.g., partition trees, canonical factor analysis).

A diagram of the LSTNR method pipeline is depicted in Figure 1. Briefly, expression levels of individual genes (Ensembl annotation) were calculated as the normalized rate of deduplicated and uniquely aligned reads per million of total sequenced reads (RPM) overlapping their annotated genomic coordinates (reference genome: hg19). Statistical tests of differential gene expression were performed using a weighed two-way ANOVA model (gene × group blocks) of log_2_-transformed fold changes (Log2FC) in RPM relative to gene-wise mean RPM either from all samples (for phenotype profiling experiments) or from a reference group (for treatment vs. control experiments) with *N* ≥ 3 replicates per group. Resolution weights of genes corresponded to the cumulative hazard of gene-wise significance scores from two-way ANOVA testing of the linear predictor of RPM from GLM of the natural parameter ***B***(**ϑ**) to an exponential continuous-valued distribution with a canonical inverse link function (Nelder and Wedderburn, 1972). Gene-wise significance of Log2FC variation based on weighed ANOVA inference testing were adjusted by the Benjamini–Hochberg method for multiple comparisons (Benjamini and Hochberg, 1995). Further details on the statistical treatment of count-level data under the LSTNR method are available in the Supplementary Methods section. All metrics and statistical analyses were carried out using JMP® 13.0.0 64-bit statistical software (SAS, Cary, NC).

### Stratification of significantly expressed genes

Significant genes (SGs) were identified as those with significantly different weighed ANOVA scores (FDR adj. *p* < 0.05); DEGs equaled the subset of SGs with a minimum practical effect size δ_Log2FC_ > δ_Effect_ and *post-hoc* pairwise-significant Log2FC differences between at least two groups (Student's *t*-test *p* < 0.05). As a reproducibility benchmark, we refer to LSTNRs as the subset of SGs in which a minimum practical effect size δ_Log2FC_ > δ_Effect_ was detected and at least one group exhibits average Log2FC signal vs. baseline greater than transcriptome-wide measurement noise (or SNR > 1) where noise is defined as the 95% Tolerance Interval (Odeh and Owen, 1980; Hahn and Meeker, 1991; Tamhane and Dunlop, 2000) of gene × group residuals among SGs. Finally, we refer to the subset of genes classified as both DEGs and LSTNRs as DEGs with reproducible expectation estimates (DEGREEs)—i.e., genes with relevant effect size, significant *post*-*hoc* pairwise differences, and prospective SNR > 1.

### Extraction of candidate genes for transcriptional profiling and biomarker analysis

To extract a list of DEGREEs with the highest transcriptional profiling potential and minimal Type II error rates, we calculated within-gene observed effect sizes (Δ_Log2FC_), as well as estimated retrospective statistical power means (≪π≫) and 95% lower confidence limits (π_low_). DEGREEs with π > 90% were listed in descending order by Δ_Log2FC_ first, and then by π_low_. The difference between successive values of Δ_Log2FC_ going down the list of ranked DEGREEs (also known as lag differences) were calculated, with the expectation that genes with higher retrospective statistical power also show larger Δ_Log2FC_ values; when true, this expectation results in a list of negative-valued lagging differences. The minimal subset of characteristic genes, or Profiler DEGREEs, corresponds to the subset of DEGREEs ranked by π_low_ that all show negative Δ_Log2FC_ lag differences before the first positive instance is found.

To obtain a reductive set of biomarker gene candidates, we performed sequential partition tree analysis (without repetition) based on the list of Profiler DEGREEs. The predictive machine-learning power of partitioned data is reported graphically via receiver operating characteristic (ROC) curves specific to each known phenotype. The minimum number of biomarkers needed for partitioning equals the number of phenotypes being partitioned minus one. Partitioning performance was then evaluated by all-at-once discriminant analysis based exclusively on the data from the biomarkers selected by sequential partitioning. Predictive machine-learning power of the canonical multivariate factors of discrimination, estimated using only biomarker data, is reported graphically via ROC curves for each expected phenotype. Phenotype segregation is depicted in multivariate space using 2-component canonical factor plots.

### Analyzed datasets

For this work, an *in silico* dataset of simulated RNAseq counts—originally assembled in the development of EPIG-seq (Li and Bushel, 2016)—was used to validate the performance of the LSTNR method. In addition, the ability of the LSTNR method to extract transcriptional signatures and expression patterns from experimental data was tested on two publicly available RNAseq data sets: one for breast cancer primary tumors under 4 breast cancer molecular subtypes deposited in The Cancer Genome Atlas (TCGA) (Cancer Genome Atlas, 2012), and another one from the MAQC phase III SEQC crowd source toxicogenomics (TGxSEQC) effort using livers of male Sprague-Dawley rats after exposure to hepatotoxic agents sharing modes of action (MOA) (Gong et al., 2014; Wang et al., 2014). Further details for each of the three datasets in this study, as well as gene annotation conventions used for each, are outlined in the Supplementary Methods section.

## Results

### EPIG-seq simulated data

**Figure 2:**
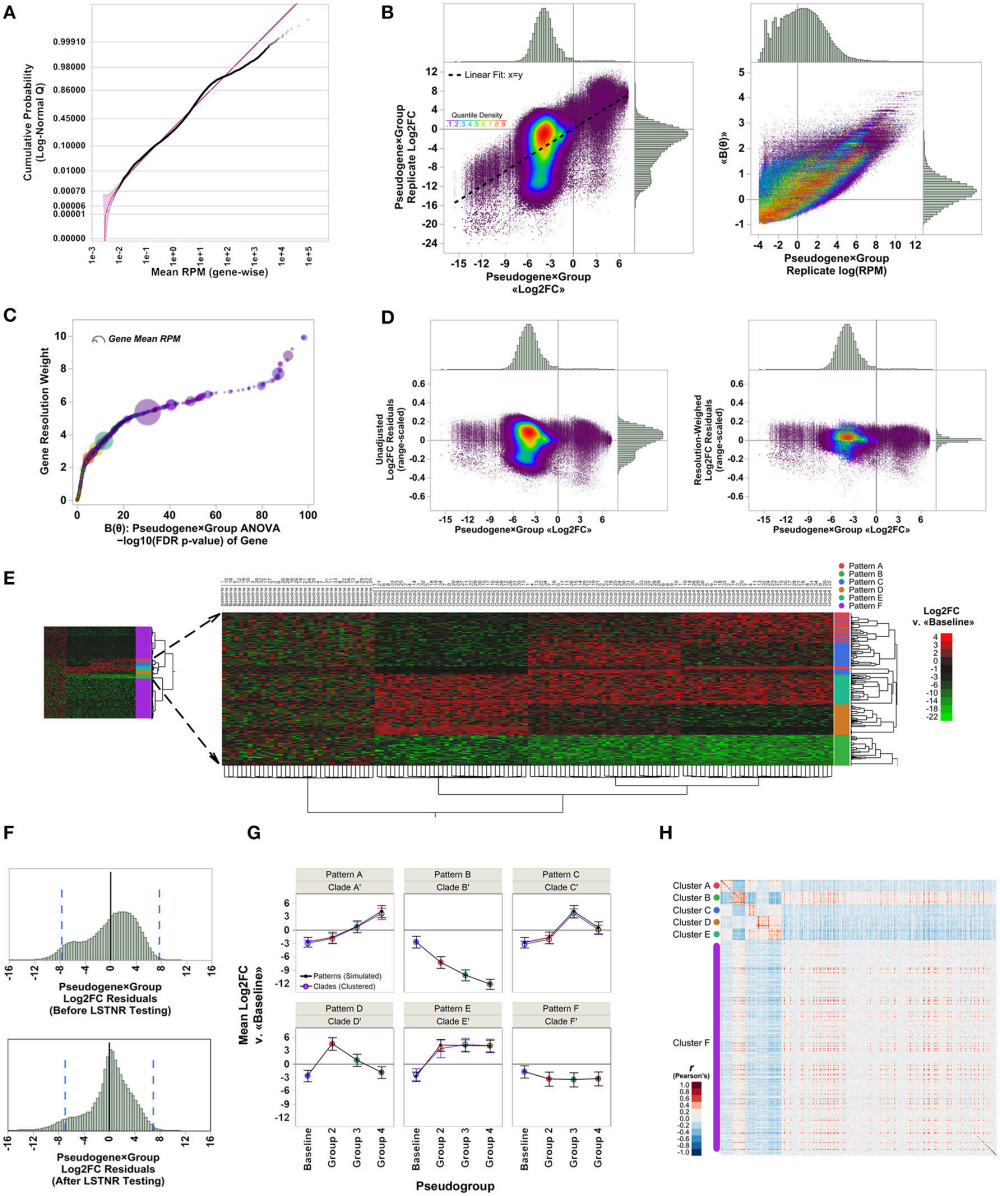
Analysis of EPIG-seq *in silico* test data by the LSTNR method. **(A)** Quantile plot of pseudogene-averaged RPM across pseudoreplicates, overlaid onto their best-fit threshold lognormal distribution parametric model (purple); shading around the parametric fit represents the simultaneous 95% confidence interval of predicted means. **(B)** Linear fitting and distribution of relative expression metrics for pseudogene × group blocks with respect to individual pseudoreplicates. Left: average of Log2FC measurements vs. Log2FC from individual pseudoreplicates; right: values of the canonical link function log (RPM) of pseudoreplicates vs. pseudogene × group block averages of the linear predictor function, ≪***B***(**θ**)≫, estimated by GLM. Colors of individual data points corresponds to the estimated quantile density of Log2FC values in mean vs. replicate space (left panel) as indicated by the adjacent color scale. **(C)** Gene resolution weights as a function of FDR-adjusted significance levels of pseudogenes as determined by pseudogene × group two-way ANOVA based on the linear predictor ***B***(**θ**). Point coloring corresponds to that in B; size of each data point is representative of pseudogene-averaged RPM across pseudoreplicates. **(D)** Distribution of range-scaled residuals around the average of pseudogene × group blocks before (left) and after (right) multiplying Log2FC measurements by gene resolution weights depicted in **(C)**; point coloring corresponds to that in **(B)**. **(E)** Heatmap plot for two-way unsupervised clustering of pseudoreplicates (horizontal) and 4,541 significant pseudogenes (FDR *p* < 0.05) detected by LSTNR analysis based on Log2FC vs. the mean RPM in the baseline group. Left: bird view of the entire heatmap; center: magnification to subset of 1,000 pseudogenes from five simulated co-expression patterns and their correspondence with inferred hierarchical clades; right: heatmap is colored on a green-black-red gradient scale of Log2FC values relative to baseline (green, downregulated; black, same; red, upregulated). **(F)** Distribution of net residuals around pseudogene × group Log2FC averages, based on 20,000 analyzed pseudogenes (before LSTNR testing) and 4,541 pseudogenes with statistically significant resolution-weighed differences (after LSTNR testing). Dotted blue lines to the left and right of the x-axis origin enclose the predicted 95% tolerance interval of residuals in each respective plot. **(G)** Average Log2FC expression ± *s.d*. of pseudogenes in simulated patterns vs. inferred clades of co-expression across groups. **(H)** Heatmap depicts Pearson's correlation coefficient *r* values between 4,541 statistically significant pseudogenes. Pseudogenes are displayed by groups of inferred co-expression clades; heatmap is colored on a blue-white-red gradient scale of Pearson's correlation coefficient *r* values (blue, negative correlation; white, not correlated; red, positive correlation). *N* = 35 pseudoreplicates in each of 4 simulated condition groups: baseline, and groups 1 to 3; number of simulated co-expression patterns: five, with 200 pseudogenes each. Simulation comprises 140 total pseudoreplicates with reads distributed among 20,000 total pseudogenes.

We used a data set of simulated RNAseq counts to validate the performance of the LSTNR method. The simulated data set was originally assembled in the development of EPIG-seq, a similarity scoring methodology for count-based data that catalogs co-expressed genes under patterns of differential expression among multiple conditions (Li and Bushel, 2016). To perform independent filtering, we fit empirically observed RPM averages from each of the 20,000 simulated pseudogenes across the entire 140-pseudoreplicate set to parametric distribution functions. We found the best fit model corresponded to a 3-parameter lognormal distribution (Figure 2A), which is equivalent to a normal distribution of RPM in logarithmic scale, with a threshold value γ representing the minimum and positive value of gene-wise average RPM supported by the fitted distribution. We reasoned the fitted threshold γ = 2.4 × 10^−3^ (95% prediction CI: 0.9 × 10^−3^-3.5 × 10^−3^) was the best candidate value to use for independent filtering across pseudogenes with simulated reads. In the case of this simulated data set, independent filtering against the threshold parameter γ did not exclude any pseudogenes from subsequent analysis—meaning all listed pseudogenes were “detectable” assuming the estimated error model of simulated read counts was properly fit by a lognormal distribution (Table 1).

**Table 1:** Step-by-step output as numbers of qualifying genes along the LSTNR analytical pipeline for a validation *in silico* dataset (courtesy of Li and Bushel, 2016; doi: 10.1186/s12864-016-2584-7).

**Criteria**	***In Silico*: EPIG-seq (*N* = 140)**
Simulated pseudogenes	20,000
Distribution of pseudogene-wise RPM means	P(y) ~ *Normal_3*P*_*(log[y];σ′,μ′,γ); *y* = RPM
	σ′ = 25.4
	μ′ = 25.4
	γ = 2.5 × 10^−3^ RPM
**Independent filtering:** pseudogenes with average y > γ	20,000
**Linearized normalizing transformant:** GLM Linear Predictor	Log[y]
**Transformant two-way ANOVA:** resolved pseudogenes across groups with respect to pseudogene-wise mean	9,492
**Resolution-weighed ANOVA:** significant pseudogenes with FDR adj. *p* < 0.05 based on differences in resolution-weighed RPM log-fold changes (Log2FC) relative to baseline group	4,541

The next step to implement the LSTNR method was to find a metric that places RPM-values from each of the detected pseudogenes (i.e., that passed independent filtering) in the context of the entire dataset to describe the relative *resolution* in detected RPM-values for each pseudogene. This can be achieved through generalized linear modeling, or GLM (Nelder and Wedderburn, 1972). In the case of the EPIG-seq *in silico* data, which followed a lognormal distribution, rewriting in exponential family form showed the linear predictor ***B*** = log(RPM) (Figure 2B). Further algebraic inspection revealed the best model to match averages of lognormally distributed ***B*** with their underlying variation is a normal distribution. Thus, transformant values of ***B*** did not require a normalizing manipulation (i.e., **η** = ***B***) going further down the LSTNR pipeline.

Transformant log(RPM) ***B***-values were tested by two-way ANOVA to estimate transformant significance scores for each pseudogene; after multiple testing adjustment by the false-discovery rate approach (Benjamini and Hochberg, 1995), we found transformant FDR *p* < 0.05 in 9,492 of the 20,000 total pseudogenes, meaning 47.46% of all pseudogenes showed variability in read counts in one or more groups that was statistically discernible (***resolvable***) from that of all pseudoreplicates combined (Table 1). We then ranked those transformant significance scores from least to most significant and calculated their position within the ranking; this is the complement of the cumulative density function, also known as the survival function. Finally, to create a metric that gives higher weight to pseudogenes detected with better resolution, we took the negative logarithm of the survival for transformant significance scores function (known as the cumulative hazard rate). This metric is smallest for pseudogenes with low resolution in RPM-values that change little across replicates, and largest for pseudogenes with RPM-values that are either very large, very variable or both (Figure 2C). We estimated these metrics and assigned them to their corresponding pseudogenes; later on, we used them as “resolution weights” to account for different resolution levels for pseudogenes based on their net read counts.

To detect significantly expressed pseudogenes, we first calculated log_2_-fold changes (Log2FC) relative to the average RPM in the baseline group for each gene; then, we combined Log2FC of genes with their resolution weights in a two-way multivariate ANOVA model. We reasoned that scaling Log2FC relative expression measurements by their resolution metric would also homogenize the scale of variation around the means of individual genes in each experimental statistical group. Indeed, we found that the relative dispersion and homogeneity of Log2FC residuals improved after scaling by gene resolution weights (Figure 2D). In all, we detected 4,541 statistically significant pseudogenes by resolution-weighed two-way ANOVA (FDR adj. *p* < 0.05). This pool of statistically significant pseudogenes contained all 1,000 differentially expressed pseudogenes with simulated co-expression patterns, as well as an extra pool of 3,541 pseudogenes from the unpatterned group exhibiting only random noise (Figure 2E).

One important aspect to consider in benchtop validation of RNAseq experiments is how well the dispersion in RNAseq output can be projected. This projection is critical to confirm RNAseq estimates by qPCR, since it helps determine both: (a) how large should RNAseq expression differences be in validation assays to be reliable; and (b) which genes are the most reliable validation candidates based on their RNAseq expression differences. With that principle in mind, we calculated 95% tolerance intervals around the mean log_2_(RPM) measurements of all 20,000 pseudogenes in the EPIG-seq simulated data set that passed independent filtering, as well as among the 4,541 statistically significant pseudogenes identified after resolution-weighed ANOVA. We found log_2_(RPM)^95%TI^ = ±7.7 among all 20,000 pseudogenes, and log_2_(RPM)^95%TI^ = ±7.0 among 4,541 statistically significant ones. In other words, assuming these data were derived from a “true” sample of biological specimens, one could project with 95% confidence that 95% of differences in expression between groups, and for any particular pseudogene, may be ~200-fold off from their “true” expected value, or ~130-fold when adjusted for noise among significant pseudogenes, due only to experimental variability between replicate experiments (Figure 2F).

The unpatterned pseudogenes with statistically significant expression levels detected through LSTNR could aggregate to excessive levels of “background noise.” This “noise” could be detrimental to statistical clustering or discriminant analyses, and may undermine the capacity to extract “true” expression patterns. To address this point, we performed naïve hierarchical clustering (Ward's method) and found that, even though unpatterned pseudogenes accounted for most of the detected differential pseudogenes overall, all differentially expressed pseudogenes were agglomerated in the hierarchical tree under simulated patterns. We also found that the 3,541 unpatterned but statistically significant pseudogenes were segregated apart from the 1,000 patterned ones (Figure 2E). Furthermore, 927 out of the 1,000 patterned differential pseudogenes were assigned into five well-separated clades of expression trends that matched the co-expression patterns prescribed *in silico* (contingency analysis Pearson's *p* < 0.0001). Altogether, the LSTNR method detected statistically significant pseudogenes belonging to both patterned and unpatterned expression trends, but did not compromise the ability to discriminate between both kinds of pseudogenes, nor their correct expression patterns, by standard clustering analyses.

Still, any benefit of implementing the LSTNR workflow is only substantial if it matches or surpasses the performance of already existing methodologies to tease out coordinated patterns of differential gene expression, such as EPIG (a pipeline tailored for microarray data) and EPIG-seq (a modified version of EPIG to handle count-based data) (Chou et al., 2007; Li and Bushel, 2016). To address this point, we assembled the confusion matrix of pseudo-gene cluster assignments per LSTNR analyses of the *in silico* dataset, and compared to those obtained by EPIG and EPIG-seq analyses (courtesy of Li and Bushel, 2016) in terms of the sensitivity (the true positive rate) and specificity (the true negative rate) of inferred cluster membership among differential pseudo-genes detected per platform (Table 2). We found the LSTNR method showed >94.5% specificity rates across simulated clusters, much like those from EPIG and EPIG-seq, thus indicating that pseudo-genes identified as differentially expressed are rarely misclassified under their originally prescribed expression patterns regardless of the chosen methodology. However, LSTNR detected more differential pseudo-genes than either EPIG or EPIG-seq, as shown by improved sensitivity rates per cluster when using LSTNR (77.5–100%) vs. either EPIG (17.5–68%) or EPIG-seq (55.5–84.5%). Put together, these results indicate that the LSTNR method successfully extracts more DEGs, all while grouping them by their true underlying expression patterns at the same or improved rates, than other similar pipelines.

**Table 2:** Confusion matrices for differential pseudo-gene pattern assignment by EPIG, EPIG-seq, and LSTNR implementation based on a validation *in silico* dataset (EPIG and EPIG-seq: courtesy of Li and Bushel, 2016; doi: 10.1186/s12864-016-2584-7.

**Method**	**Clustered Clades**	**Simulated Expression Pattern**					**Total**	**Sensitivity (%)**	**Specificity (%)**
		**Pattern A**	**Pattern B**	**Pattern C**	**Pattern D**	**Pattern E**			
EPIG	Group A	0	0	46	0	0	46	23	100
	Group B	0	0	0	0	136	136	68	100
	Group C	0	39	0	0	0	39	19.5	100
	Group D	0	0	0	60	0	60	30	100
	Group E	35	0	0	0	0	35	17.5	100
EPIG-Seq	Group A	7	132	11	0	0	150	66	98.2
	Group B	0	0	169	0	0	169	84.5	100
	Group C	135	26	5	0	0	166	67.5	96.9
	Group D	0	0	0	15	166	181	83	98.5
	Group E	9	23	0	111	43	186	55.5	92.5
LSTNR	Clade A'	177	0	12	0	4	193	88.5	98.4
	Clade B'	0	200	0	0	0	200	100	100
	Clade C'	1	0	155	0	1	157	77.5	99.8
	Clade D'	0	0	0	200	0	200	100	100
	Clade E'	22	0	33	0	195	250	97.5	94.5

Among the 927 differential pseudogenes correctly assigned to simulated patterns of expression detected by the LSTNR method, 400 were classified into clades that matched their *in silico* counterparts in full (patterns B and D); in contrast, the EPIG-seq method matched pseudogenes completely to their simulated pattern only for B. The five hierarchical clades identified by LSTNR also matched their respective *in silico* patterns in terms of average expression levels within statistical groups (Figure 2G). We found similar pattern-detection performance among the 4,541 statistically significant pseudogenes detected with the LSTNR method when clustering them by the Pearson product-moment correlation scores of their Log2FC vs. the baseline average (Figure 2H). In sum, the LSTNR method not only identified the five simulated expression patterns just like the EPIG-seq pipeline, but did so by assigning differential pseudogenes to their correct patterns with greater accuracy, by routing read count data to traditionally robust statistical tests (e.g., multivariate ANOVA), and without relying on user-defined parameters.

### Breast cancer classification by hierarchical clustering of LSTNR-detected DEGs

Next, we used RNAseq data from breast ductal carcinoma biopsies deposited in The Cancer Genome Atlas (TCGA) to test the LSTNR method. The data set collected for this assessment consisted of 160 primary tumors classified under 4 breast cancer molecular subtypes groups in equal sample sizes (luminal A, luminal B, Her2-enriched and basal-like) and 40 matching normal breast tissue biopsies as controls (Cancer Genome Atlas, 2012). Besides consisting of patient-derived data that is clinically relevant, this collection of TCGA specimens presents some practical and common challenges associated with clinical data, including high variability between patients and between cohorts from an epidemiological perspective, and batch effects from a technical one. All these challenges, which severely undermine significance testing, have clinical consequences: if the only solution to understand the performance of RNAseq as a diagnostic tool is to design large patient cohorts, it risks rendering RNAseq too expensive or slow to be useful in a clinical setting. Here, our goal was to implement the LSTNR method and define how successful it was in sorting out the correct breast cancer molecular subtypes (and their transcriptional signatures) based on a much smaller cohort of patient-derived specimens than the TCGA database itself.

We analyzed TCGA dataset following two approaches: (a) we split the specimens from each of the four molecular subtypes into four independent and mutually exclusive sample subsets (or *realizations*) of equal size, performed parallel statistical analyses for each, and identified the subset of DEGs detected in common by all four separate per-realization analyses; and (b) we analyzed the entire dataset all at once in a single run. Altogether, this strategy comprised a total of 5 separate implementations of the LSTNR method on patient-derived breast cancer data: four per-realization analyses and one single-shot analysis. By following this analytical design, we sought to establish the degree of observed concordance between DEGs detected by a single-shot analysis vs. the consensus from multiple tests on subsampled statistical groups. We also aimed at determining whether capturing more DEGs with an all-encompassing single-shot analysis is superior in quality and performance to performing split tests in tandem and extracting a consensus DEG list.

**Figure 3:**
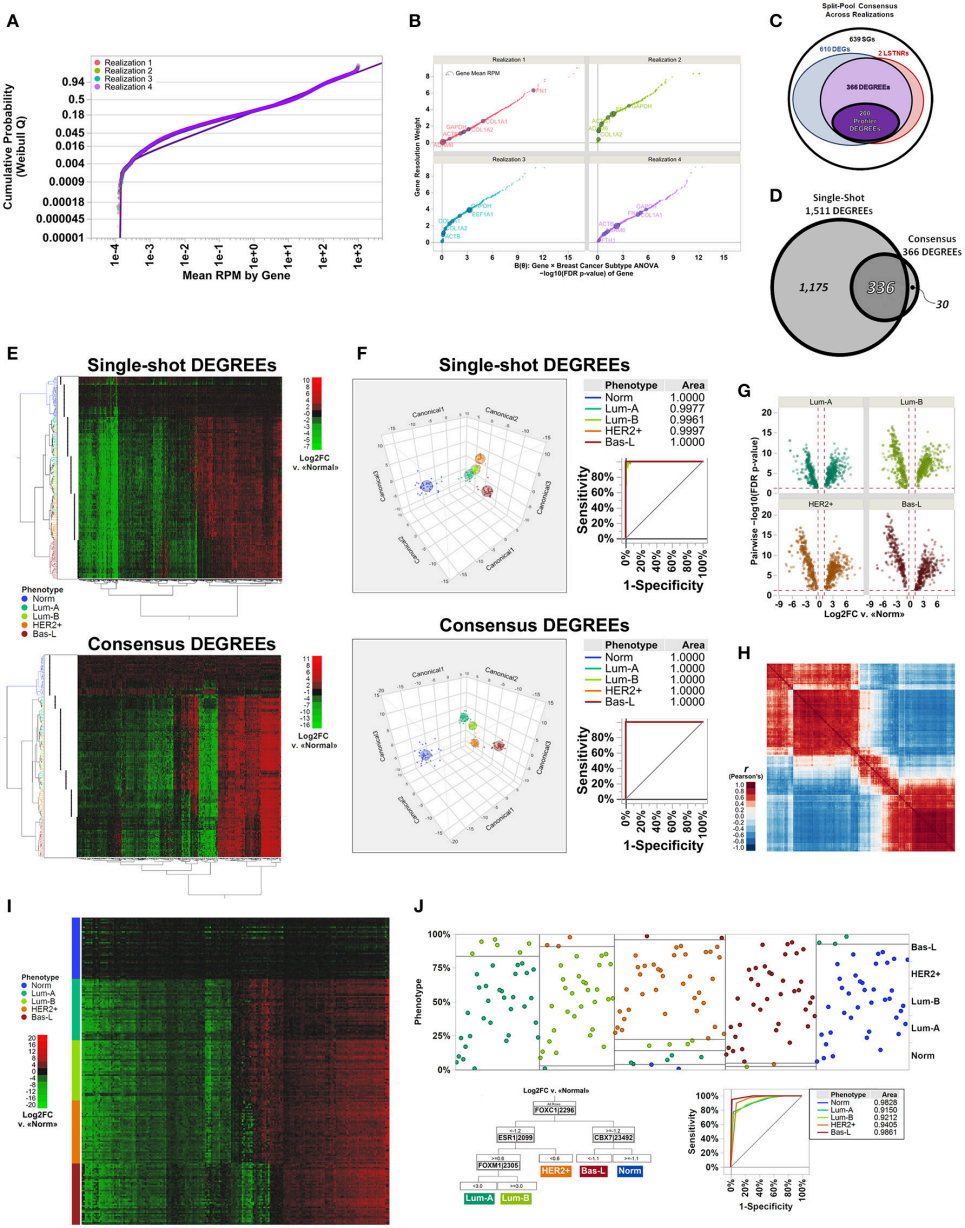
Molecular subtype discrimination of transcriptional signatures from patient-derived breast cancer specimens using the LSTNR method by split-pool and single-shot approaches. **(A)** Quantile plot of gene-averaged RPM across replicates, overlaid onto best-fit threshold Weibull distribution parametric models per individual realization; fit lines and points are colored by the realization they were tested under. **(B)** Gene resolution weights calculated within each realization, as a function of FDR-adjusted significance levels of genes, based on linear predictor ***B***(**θ**) gene × group two-way ANOVA. Point coloring indicates realization tested; size of each data point is representative of gene-averaged RPM across replicates within each realization; also, the top 10 genes found in all realizations with highest RPM scores, and their identities, are shown with dark outlines. **(C)** Venn diagram depicting the number of shared gene symbols among consensus SGs, DEGs, LSTNRs, and DEGREEs identified in all realizations. **(D)** Venn diagram depicting the concordance between DEGREEs detected by all-at-once single-shot analysis of the TCGA dataset vs. the set of consensus DEGREEs identified across separate realizations. **(E)** Heatmap plots for two-way unsupervised clustering of all specimens (horizontal) and DEGREEs detected by single-shot analysis (top) or consensus across realizations (bottom) based on differential expression levels. Coloring of row dendrograms and labels represent the phenotype as annotated in TCGA database for each specimen. Dot columns on the left of heatmap plots depict inferred specimen clades via unsupervised clustering of depicted genes in each heatmap separately. Right: heatmaps are colored on green-black-red gradient scales of Log2FC values relative to baseline (green, downregulated; black, same; red, upregulated). **(F)** Three-factor discriminant analysis and plots of tested specimens based on Log2FC measurements of single-shot DEGREEs (top) vs. consensus DEGREEs (bottom). Coloring of lines and points in depicted plots represent the phenotype as annotated in TCGA database for each specimen. ROC curves and their AUC values are also shown. **(G)** Volcano plots of 200 Profiler DEGs across all breast cancer subtype specimens relative to normal group; y-axis: *post*-*hoc* pairwise significance scores of Log2FC measurements; x-axis: mean Log2FC vs. mean RPM of normal specimens. **(H)** Heatmap depicts Pearson's correlation coefficient *r*-values between 200 Profiler DEGREEs. Order of Profiler DEGREEs corresponds to their arrangement by unsupervised clustering, as shown in **(E)**. Heatmap is colored on a blue-white-red gradient scale of Pearson's correlation coefficient *r*-values (blue, negative correlation; white, not correlated; red: positive correlation). **(I)** Heatmap plots of all breast cancer specimens sorted by phenotype and replicate number (rows) vs. Profiler DEGREEs detected by consensus across realizations (columns) based on differential expression levels. Order of Profiler DEGREEs corresponds to their arrangement by unsupervised clustering, as shown in **(E,H)**. Coloring of row labels represent the phenotype as annotated in TCGA database for each specimen. Right: heatmap is colored on a green-black-red gradient scale of Log2FC values relative to normal specimens (green, downregulated; black, same; red, upregulated). **(J)** Inferred diagnostic biomarkers of breast cancer subtypes by sequential partitioning tree analysis of Profiler DEGREEs. Coloring of lines and points in depicted plots represent the phenotype as annotated in TCGA database for each specimen. Partition analysis ROC curves and their AUC values are also shown. *N* = 40 independent specimens per breast cancer molecular subtype: normal, luminal A, luminal B, HER2+, and basal-like. Specimen classification as annotated in TCGA database. TCGA dataset comprises 200 total individual specimens with reads aligned onto 20,532 genes overall, analyzed as a whole (single-shot) or evenly split into four separate and mutually exclusive realizations (split-pool). Reference genome: hg19.

A total of 20,532 annotated genes (hg19) with uniquely aligned reads were represented in any one of the specimens used to test the LSTNR method. Empirically observed RPM averages within realizations were fit to parametric distribution functions independently. In all cases, the best-fit model for within-gene RPM averages corresponded to a 3-parameter Weibull distribution function P(y) ~ Weibull_3P_(y;α,ß,γ) where y = RPM (Figure 3A); the estimates for the threshold parameter γ in each separate realization ranged between 1.5 × 10^−3^-11 × 10^−3^ RPM (Table 3). The threshold parameter γ, representing the minimum average RPM-value explained by the parametric Weibull fit, was added across the dataset to circumvent arithmetic issues with zero-valued data when estimating relative expression ratios.

**Table 3:** Step-by-step output as numbers of qualifying genes along the LSTNR analytical pipeline for RNAseq data deposited in TCGA from four realizations of patient-derived breast cancer transcriptomes across four molecular subtypes (courtesy of Li and Bushel, 2016; 10.1186/s12864-016-2584-7).

**Criteria**	**Breast Cancer Molecular Subtypes (TCGA) (*N* = 200)**				
	**Realization 1 (*N* = 50)**	**Realization 2 (*****N*** **=** **50)**	**Realization 3 (*****N*** **=** **50)**	**Realization 4 (*****N*** **=** **50)**	**All Specimens (*****N*** **=** **200)**
Genes with uniquely aligned reads	20,532				
Distribution of gene-wise RPM means	P(y) ~*Weibull_3*P*_*(y;α,ß,γ); y = RPM				
	α = 25.4 RPM	α = 24.1 RPM	α = 25.0 RPM	α = 21.9 RPM	α = 22.2 RPM
	ß = 0.53	ß = 0.53	ß = 0.54	ß = 0.49	ß = 0.49
	γ = 9.9 × 10^−3^ RPM	γ = 6.6 × 10^−3^ RPM	γ = 1.1 × 10^−2^ RPM	γ = 1.6 × 10^−3^ RPM	γ = 1.5 × 10^−3^ RPM
**Independent filtering:** Genes with average y > α	*8,005*	*8,110*	*8,083*	*8,562*	8,538
**Linearized normalizing transformant:** GLM Linear Predictor	(y–γ)^−1^				
**Transformant two-way ANOVA:** resolved genes across groups with respect to gene-wise mean	*4,295*	*381*	*638*	*2,281*	2,851
**Resolution-Weighed ANOVA:** Significant Genes (SGs) with FDR adj. *p* < 0.05 based on differences in resolution-weighed RPM log-fold changes (Log2FC) relative to baseline condition	*4,465*	*5,086*	*4,537*	*4,618*	6193
	*Altogether: 7,749*				**Final Overlap: 1,509**
	*Intersection: 1,617*				
**Differential expression:** DEGs = subset of SGs that exhibit both: resolution-weighed effect size above 5% of gene-wise variation (δ_Log2FC_ > 0.3 × σ_SSR_); and *post*-*hoc* pairwise-significant Log2FC differences between at least two groups (Student's t-test p < 0.05)	*3,736*	*3,377*	*3,497*	*3,617*	6,093
	*Altogether: 6,407*				**Final Overlap: 908**
	*Intersection: 976*				
**Reproducibility:** LSTNRs = subset of SGs that exhibit both: resolution-weighed effect size above 5% of gene-wise variation (δ_Log2FC_ > 0.3 × σ_SSR_); and at least one group with Log2FC differences vs. baseline greater than 95% Tolerance Interval of gene × group residuals among SGs (*post*-*hoc* pairwise-significance not required)	*1,370*	*1,102*	*1,130*	*1,210*	1,511
	*Altogether: 2,193*				**Final Overlap: 337**
	*Intersection: 368*				
**Expectable DEGs:** DEGREEs = Ensembl-annotated DEGs with a reproducible expectation estimate (i.e., DEGs that are also LSTNRs) and official Entrez symbol	*Intersection: 366*				1,511
					**Final Overlap: 336**
**Transcriptional profiling:** Profiler DEGREEs = top DEGREEs ranked by retrospective statistical power with monotonically decreasing within-gene effect sizes Δ_Log2FC_	**200 Profiler DEGREEs (consensus)**				
**Diagnostic targets:** Biomarkers = minimal subset of Profiler DEGREEs with predictive discriminant power based on sequential partition tree analysis (ROC scores > 0.9 per phenotype)	CBX7, ESR1, FOXC1, and FOXM1				

It is well-known that, for GLM, Weibull distributions with fixed shape parameter ß can be restated by simple algebraic substitution in the form of an exponential distribution, for which the linear predictor ***B*** ~ (y–γ)^−1^ and the normalizing link function **η** ~ 1/***B*** (Nelder and Wedderburn, 1972; Aitkin and Clayton, 1980). Therefore, to determine the resolution level of detected reads per gene, we calculated transformant ***B***-values from within-gene RPM averages, normalized their distribution by using their reciprocal values, and tested them by multivariate ANOVA (see Supplementary Methods for details). Analysis of the entire dataset at once also identified, with parameters almost equal to the ones estimated from realizations, a 3-Parameter Weibull distribution as the best parametric fit (Table 3).

The number of retained genes following independent filtering with respect to the Weibull scale parameter α (Table 3) was about the same in all five analyses (8,005–8,562 across realizations; 8,538 single-shot), yet the estimated number of significantly resolved genes, defined by genes with transformant FDR *p* < 0.05, was sensitive to the realization they came from. The number of resolved genes varied by up to one order of magnitude (e.g., 381 genes in realization 2 and 4,295 genes in realization 1). Even though the number of resolved genes estimated by single-shot analyses (2,851 total with transformant FDR *p* < 0.05) fell within the order of magnitude resolved genes by the other four per-realization analyses, it was still over 50% larger than the average of 1,899 resolved genes per realization (Table 3). These findings illustrate that the independent filtering criteria used to exclude poorly represented genes were equally valid whether they were calculated from a subset or from the entire set of individual specimens in an experiment. In contrast, estimates of the dynamic range of sequencing representation are off-target when using different data subsets. Therefore, expecting independent RNAseq experiments to detect the same pool of significantly resolved genes is sensible only when the replicate sets in one experiment are statistically indistinguishable from the replicate sets in another—an expectation that is grossly misguided among patient-derived specimens.

Significance scores of estimated RPM transformants are only as good as the samples they reflect, and will change depending on whether variation within statistical groups is more or less heterogeneous for different subsets. We premised that each of the subsets is representative of the entire set, and that ranking of the significance scores of genes should be similar across subsets no matter their actual *p*-values; that was the case when we calculated resolution weights of genes per realization (Figure 3B). Interestingly, we found that many genes at the high-end of the resolution weight curve tended to show high read count numbers, yet many genes with high RPM averages showed poor significance scores for their transformant ***B***-values. These results suggested that average RPM of genes, though likely to capture finer differences, were not reliable predictors of gene expression resolution.

We then performed resolution-weighed multivariate ANOVA tests of Log2FC differences relative to the average of normal tissues, either per realization or single-shot. We found that, by including resolution scaling, the number of detected significant genes (SGs) became more consistent: between 4,465 and 5,086 SGs from the four independently analyzed realizations, and 6,193 SGs by the single-shot analysis (Table 3; see Table S1 for gene symbols). These results were striking because the resolution scaling strategy of the LSTNR method effectively stabilized the number of detected SGs across all five separate tests, even though the number of genes with significant transformant variation (FDR *p* < 0.05) were quite different in each of these separate analyses (as mentioned earlier).

In all, we found a common pool of 1,617 SGs across all four realizations (Table 3; see Table S1 for gene symbols), corresponding to 32–36% concordance rates with respect to the total SGs in each one separately (range of SGs per realization: between 4,465 and 5,086). Those observed concordance rates carried down to increasingly stringent levels of stratified practical significance across separate realization tests (see section Materials and Methods and Supplemental Materials for further details): from the pool of 1,617 common SGs from per-realization analyses, we detected 976 DEGs (vs. number of DEGs between 3,377 and 3,736 in separate realizations; 26–29% concordance rates), and 368 LSTNRs (vs. number of LSTNRs between 1,102 and 1,370 in separate realizations; 27–33% concordance rates). Overall, between DEGs and LSTNRs, we found an intersecting set of 366 genes (37% of DEGs), which we termed DEGs with reproducible expectation estimates (DEGREEs) because they: (a) were statistically significant when adjusted for sequencing resolution; (b) showed Log2FC variation between groups greater than a reference 5% practical effect size; (c) exhibited *post*-*hoc* pairwise significant differences in Log2FC between groups; and (d) exhibited Log2FC differences with SNR > 1 relative to transcriptome-wide Log2FC measurement error (Figure 3C). Likewise, refining the pool of 6,193 single-shot SGs with additional stringency resulted in 6,093 DEGs and, among them, 1,511 LSTNRs all contained within the DEG pool. Hence, all 1,511 LSTNRs (or 25% of DEGs) were also DEGREEs (Table 3; see Table S1 for gene symbols).

Next, we interrogated whether detected SGs were similar between per-realization and single-shot analyses at different levels of statistical stringency. Of the 1,617 common SGs across realizations, 1,509 (over 93%) were also among the 6,193 SGs detected in the single-shot analysis using the entire data set. This means that about three out of four (75.6%) of all SGs identified by single-shot testing of the entire dataset are not replicated in parallel tests of mutually exclusive subsets of samples from the same experiment. We then evaluated the concordance rates between intersecting genes across per-realization vs. single-shot analyses, and detected final overlaps of 908 DEGs (93% concordance w.r.t. common per-realization pools; 15% concordance w.r.t. single-shot pool), 337 LSTNRs (92% concordance w.r.t. common per-realization pools; 22% concordance w.r.t. single-shot pool), and 336 DEGREEs overall (92% concordance w.r.t. common per-realization pools; 22% concordance w.r.t. single-shot pool) (Figure 3D and Table 3; see Table S1 for gene symbols).

We also asked to what extent using DEGREEs as a gene set of choice for transcriptome-based phenotype segregation was adequate, and whether using a consensus minimal set was superior than using a larger set of DEGREEs extracted from a single-shot differential expression analysis. To do this, we performed two-way unsupervised hierarchical clustering (Ward method) of the entire set of 200 specimens based on Log2FC expression differences vs. normal breast biopsies with: (a) 366 consensus DEGREEs across all per-realization analyses; and (b) the 1,511 DEGREEs identified from the single-shot analyses. We found that unguided sorting of specimens in hierarchical clusters was in agreement with the known molecular classification of the specimens, irrespective of the set of DEGREEs used (Figure 3E). An alternative test, discriminant analysis, also showed that separation of specimens in multifactorial space was highly predictive of the correct phenotype. Surprisingly, the same tests also revealed that discrimination and predictive power both were superior when using the consensus set of 366 DEGREEs (0% misclassified specimens) instead of the larger set of 1,511 DEGREEs from single-shot analysis (3.5% misclassified specimens) (Figure 3F). This outcome suggests that the LSTNR method not only extracted the same phenotype groups by splitting specimens into parallel data subsets and extracting a consensus DEG set rather than using the entire cohort at once, but profiled them successfully using far fewer genes and specimens, and with greater accuracy, than ever reported (Perou et al., 2000; Sørlie et al., 2001; Cancer Genome Atlas, 2012).

The objective of collecting clinical data from large cohorts, as those in TCGA, is to determine what are the most reliable diagnostic signatures that distinguish closely related diseases. Thus, we tested the ability to discriminate breast cancer subtypes when using the smallest possible set of genes with reproducible expression differences that we detected by LSTNR. To that end, we selected a minimal set of 200 consensus DEGREEs that exhibited, simultaneously, the largest within-gene retrospective statistical power (≪π≫ >90%) and within-gene effect size Δ_Log2FC_; we termed these Profiler DEGREEs (Table 3; see Table S1 for gene symbols). We observed >1.66-fold statistically significant *post*-*hoc* pairwise absolute differences (*p* < 0.05) in at least one of the four breast cancer subtypes vs. normal tissue biopsies in each of the 200 Profiler DEGREEs (Figure 3G), which also showed highly correlative associations (Figure 3H). The increased statistical strength of Profiler DEGREEs over all other sequenced genes was sufficient to distinguish different expression patterns among the 200 breast tissue specimens by ordering them by subtype (Figure 3I).

Finally, to extract a minimal set of diagnostic biomarkers, we performed sequential partition tree analysis and defined what subset of genes among the 200 Profiler DEGREEs had the highest discriminative power. We found that breast cancer subtypes could be assigned with >90% diagnostic accuracy, as indicated by the area under the ROC curve of the partition tree, by differential expression analysis with respect to a normal breast tissue reference using only 4 genes: CBX7, ESR1, FOXC1, and FOXM1 (Figure 3J). Of the four biomarkers we detected, ESR1 is the only one in common with the reported signature for luminal breast cancers (ESR1, GATA3, FOXA1, XBP1, and cMYB) (Cancer Genome Atlas, 2012). Notably, only ESR1, GATA3, and XBP1 are also present in our list of Profiler DEGREEs. The fact that we detected closely related transcription factors other than FOXA1 and cMYB specifically, i.e., four members from the FOX family (FOXC1, FOXM1, FOXN3, FOXO1) and one from the MYB family (MYBL2), lends confidence to our analysis, which captured the same underlying biological mechanisms that distinguish each breast cancer subtype. However, our analysis achieved the same results with a considerably smaller cohort (*N* = 200) than the one reported previously by the TCGA consortium (*N* = 825) (Cancer Genome Atlas, 2012).

In all, these results confirm that single-shot analyses of differential gene expression yields higher numbers of detected significant differences as sample size grows. However, they also suggest that statistical testing of excessively large sample sizes at once comes at the expense of experimental reproducibility, regardless of statistical stringency. Instead, data splitting into modestly sized subsets of samples for parallel statistical analysis can extract a minimal set of representative DEGREEs shared among parallel per-realization tests. We showed that the resulting consensus DEGREE set shows a higher chance of reproducibility. Furthermore, split data processing allows for multi-threaded computation, which in principle accelerates data analysis speed and performs at a much more accessible computational footprint than a single-shot analysis.

Most importantly, a minimal consensus set of DEGREEs deduced by parallel subsampled analyses is not only more statistically powerful in theory, but also more useful in practice. Because any inferred biomarkers need to be experimentally validated, diagnosing between disease phenotypes by gene expression assays is more amenable and affordable at the bench when the number of targets is kept at their fewest; it is also less prone to practitioner's mistakes, and easier to translate to clinical practice. In the case of breast cancer subtypes, the LSTNR method excelled in extracting a theoretical minimum number of diagnostic biomarkers (a total of 4) necessary to discriminate among 4 predetermined subtypes. In practice, diagnostic testing can be carried out to validate findings, to confirm observations by other researchers, or for clinical purposes where sample numbers are limiting. Our data indicate using the 4 biomarkers detected by LSTNR to diagnose breast cancer subtypes would be at least as reproducible as using 50-gene subtype predictor microarrays reported previously (Parker et al., 2009)—only much cheaper.

### Discrimination of hepatotoxic MOAs following chemical exposure in rats

The LSTNR method, like many others, can discriminate among disease conditions with known molecular signatures (such as breast cancer) in spite of the heterogeneity in the gene expression profiles among patients because the transcriptional signatures are often overt. In the case of specimens deposited in TCGA, the evidence supporting the classification of each specimens is a combination of both histopathological criteria and transcriptional profiling, an approach pioneered almost two decades ago and refined since by Perou and others based on microarray data (Perou et al., 2000; Sørlie et al., 2001; Parker et al., 2009). This means that, although successful in phenotyping breast cancer subtypes, LSTNR implementation in the context of breast cancer subtypes is somewhat recursive.

The same cannot be said when characterizing transcriptional signatures from data generated by toxicogenomics experiments, in which healthy specimens are exposed transiently to chemical insults and assayed soon after to understand which genes respond to the exposure, and to what extent their response is coordinate or follows particular signaling pathways (also known as the mode of action, MOA). Usually, these studies use large sample sizes, but that is often the case because specimens tested for the same MOA are often exposed to more than one eliciting chemical agents, each with minimal sample sizes. In the end, this means most toxicogenomics datasets cannot be split into realization subsets, and so they must be inspected with single-shot analyses. For all these reasons, identifying MOA from toxicogenomics studies can be more challenging than transcriptional profiling of disease phenotypes.

To assess the performance of LSTNR for toxicogenomics analyses, we used a MOA training RNAseq dataset generated through the MAQC phase III SEQC crowdsource toxicogenomics effort (TGxSEQC). The TGxSEQC training dataset consists of liver transcriptomes from male Sprague-Dawley rats following exposure to hepatotoxicants (Gong et al., 2014; Wang et al., 2014). The experimental designed included five known modes of action, each induced by exposure to three different chemical agents unique to each MOA, with a replicate size of *N* = 3 per combined MOA × Agent group. In all, the experimental design comprised 45 individual specimens equally split among 15 different agents and stratified under 5 MOAs with *N* = 9. Because the purpose of this particular TGxSEQC crowdsourced experiment was to create a benchmark training set to attest performance of newly developed statistical pipelines, additional levels of complexity were introduced in the experimental design by controlling their exposure scheme: some of the chemical agents were supplied by intraperitoneal injections, and others by oral gavage using either nutritive (corn oil) or non-nutritive (water) delivery vehicles. In addition to the 45 agent-exposed specimens, an additional set of 9 livers was also collected as a control group from male rats that underwent mock treatments by different combinations of exposure schemes (i.e., intraperitoneal vs. oral gavage, and corn oil vs. water as oral vehicles).

The TGxSEQC dataset consisted of 30,852 RefSeq-annotated transcripts (rn6 reference genome) uniquely aligned reads, which we refer to as “genes” hereafter for simplicity. The best-fit model to the average RPM-values within annotated transcripts across all 54 individual liver specimens was a 3-parameter Weibull distribution function P(y) ~ Weibull_3P_(y;α,ß,γ) where y = RPM. We then performed independent filtering with respect to the Weibull scale parameter α = 6.7 RPM and retained 9,593 transcripts for differential expression analysis (Table 4). Next, to determine the resolution level of detected reads per gene, we calculated transformant ***B***-values from within-gene RPM averages by GLM with linear predictor **B**~(y–γ)^−1^ and normalizing link function **η** ~ 1/***B*** as appropriate for fixed-ß Weibull distributions (Nelder and Wedderburn, 1972; Aitkin and Clayton, 1980). Then, transformant ***B***-values were tested by gene × MOA multivariate ANOVA, i.e., irrespective of the chemical agent, route, or nutritional status of the vehicle of each specimen. We identified 3,975 significantly resolved genes, based on a transformant ***B*** FDR *p* < 0.05 criterion (Table 4). Gene resolution weights were estimated from the cumulative hazard rate of the gene × MOA multivariate ANOVA significance scores of ***B***. Also, we added the threshold parameter γ = 2.5 × 10^−3^ RPM to each individual replicate across all transcripts, and estimated Log2FC differences relative to the average of mock-treated controls. Then, we performed a resolution-weighed gene × MOA multivariate ANOVA of Log2FC differences. We detected a total 5,983 SGs; among them, we found 5,864 DEGs and, of those, 1,953 were also LSTNRs. The list of LSTNRs included 386 non-protein encoding transcripts based on their RefSeq annotation (i.e., XR_, XM_, or NR_ accession prefix), which we discarded from subsequent analysis. The remaining 1,567 LSTNRs corresponding to mRNA transcripts (NM_ accession prefix) were consolidated into a final list 1,510 DEGREEs with official non-duplicate Entrez gene symbols. Then, after comparing the ranks of retrospective statistical power and within-DEGREE effect size for Log2FC measurements across MOA groups, we identified a minimal set of 65 Profiler DEGREEs for transcriptional profiling (Table 4; see Table S2 for gene symbols).

**Table 4:** Step-by-step output as numbers of qualifying genes along the LSTNR analytical pipeline for liver transcriptomes from male Sprague-Dawley rats after toxicant exposure based on the mode-of-action training RNAseq dataset by the MAQC phase III SEQC crowdsource toxicogenomics (TGxSEQC) effort (GEO accession number: GSE55347).

**Criteria**	**Hepatotoxicity: Mode-of-Action Rat Models (*N* = 54)**
Genes with uniquely aligned reads	30,852
Distribution of gene-wise RPM means	P(y)~*Weibull_3*P*_*(y;α,ß,γ); y = RPM
	α = 6.7 RPM
	ß = 0.38
	γ = 2.5 × 10^−3^ RPM
**Independent filtering:** Genes with average RPM y > α	9,593
**Linearized normalizing transformant:** GLM Linear Predictor	(y–γ)^−1^
**Transformant two-way ANOVA:** resolved genes across groups with respect to gene-wise mean	3,975
**Resolution-Weighed ANOVA:** Significant Genes (SGs) with FDR adj. p < 0.05 based on differences in resolution-weighed RPM log-fold changes (Log2FC) relative to baseline condition	5,983
**Differential expression:** DEGs = subset SGs that exhibit both: resolution-weighed effect size above 5% of gene-wise variation (δ_Log2FC_>0.3 × σ_SSR_); and *post*-*hoc* pairwise-significant Log2FC differences between at least two MOAs (Student's t-test *p* < 0.05)	5,864
**Reproducibility:** LSTNR genes = subset of SGs that exhibit both: resolution-weighed effect size above 5% of gene-wise variation (δ_Log2FC_>0.3 × σ_SSR_); and at least one group with Log2FC differences vs. baseline greater than 95% Tolerance Interval of gene × group residuals among SGs (*post*-*hoc* pairwise-significance not required)	1,953
**Expectable DEGs:** DEGREEs = Ensembl-annotated DEGs with a reproducible expectation estimate (i.e., DEGs that are also LSTNRs) and official Entrez symbol	1,510
**Transcriptional profiling:** Profiler DEGREEs = top DEGREEs ranked by retrospective statistical power with monotonically decreasing within-gene effect sizes Δ_Log2FC_	**65 Profiler DEGREEs**
**Diagnostic targets:** Biomarkers = minimal subset of Profiler DEGREEs with predictive discriminant power based on sequential partition tree analysis (ROC scores>0.9 per phenotype)	Ucp3, Tmem86b, Sugct, Acaa1b, Hadhb, Tfam, Acaa1a, and Gsdmd

**Figure 4:**
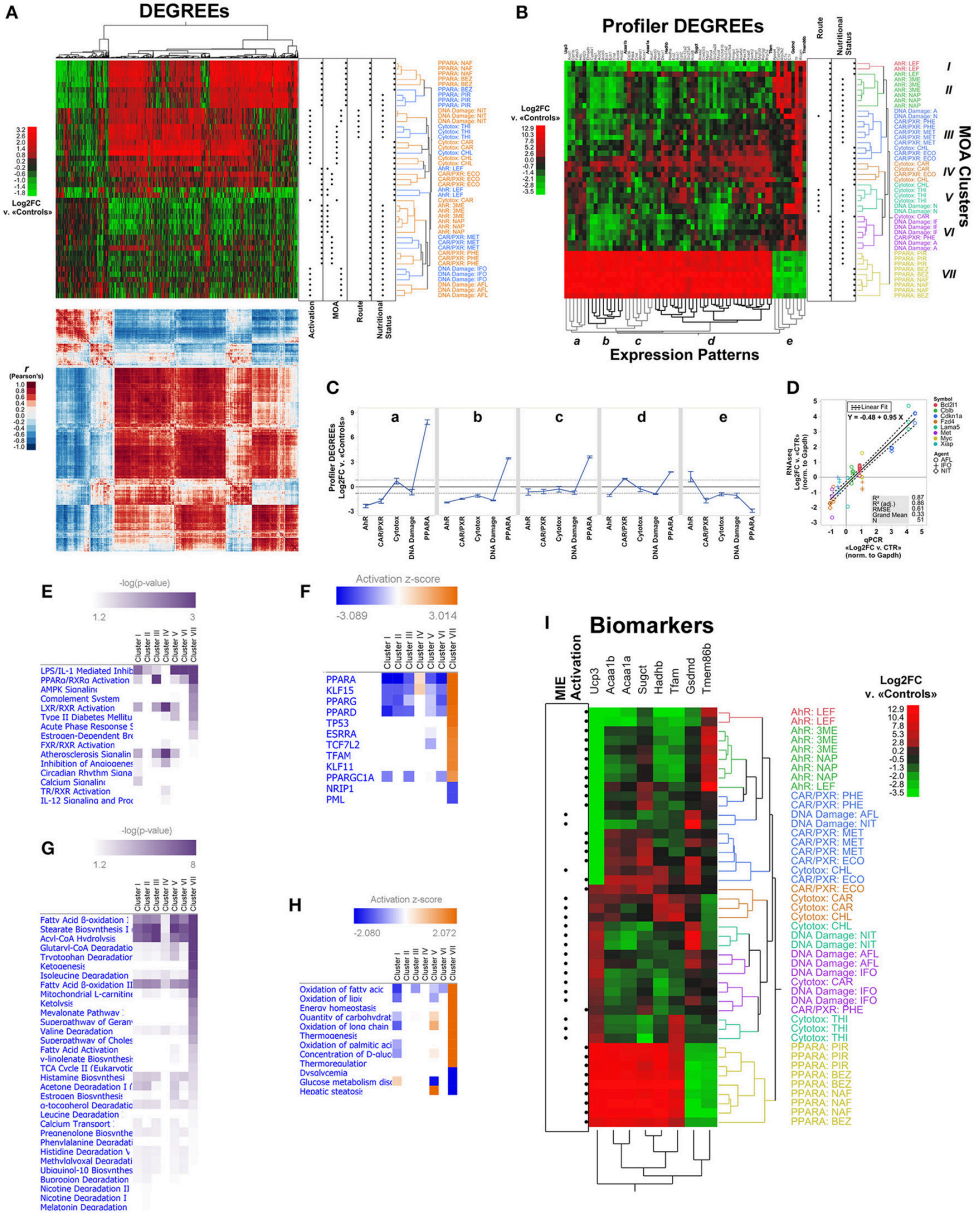
LSTNR method analysis of the MOA training RNAseq dataset by the TGxSEQC crowdsource effort. **(A)** Heatmap plots of expression differences (top) and Pearson's *r* correlation coefficients (bottom) for 45 Sprague-Dawley rat liver specimens exposed to toxic agents classified under 5 different MOAs (rows; *N* = 9 per MOA) and 1,510 DEGREEs (columns) based on Log2FC measurements vs. the average of 9 mock-treated controls. The expression heatmap of DEGREEs (top) is colored on a green-black-red gradient scale (left) of Log2FC values (green downregulated; black, same; red, upregulated); the correlation heatmap of DEGREEs (bottom) is colored on a blue-white-red gradient scale (left) of Pearson's correlation coefficient *r*-values (blue, negative correlation; white, not correlated; red, positive correlation). Order of DEGREEs in both heatmaps (columns) is illustrated by the column dendrogram in the expression heatmap, and was based on two-way unsupervised hierarchical clustering of DEGREEs and specimens based on Log2FC expression differences (expression heatmap, top). Dot plots on the right of the expression heatmap (top) depict experimental classifications of treated specimens based on: means of transcriptional activation of MIEs (left to right: non-receptor mediated, or NRM vs. receptor-mediated or RM); MOA (left to right: Ahr, CAR/PXR, Cytotoxic, DNA damage, PPARA); route of exposure (left to right: intraperitoneal injection, or oral gavage); and nutritional status of vehicle (left to right: not nutritive solution, or nutritive oil-based vehicle). Inferred specimen clades, represented in the expression heatmap (top) by row dendrograms and their respective MOA × Agent labels, are shown with alternating orange and blue coloring for clarity. **(B)** Expression heatmap for two-way unsupervised clustering of Log2FC differences, as in **(A)**, using only 65 Profiler DEGREEs (columns). Profiler DEGREE columns are labeled by their respective Entrez gene symbol (top); the names of 8 biomarkers selected from the set of Profiler DEGREEs by sequential partition tree analysis are shown raised and in bold. Coloring of the row dendrogram and its labels (left) represent inferred grouping of treated specimens into 7 MOA clusters (I-VII). Inferred co-expression patterns represented by the column dendrogram (bottom; a–e) are shown with alternating gray and black coloring for clarity. Top left: heatmap is colored on a green-black-red gradient scale (top left) of Log2FC values relative to mock-treated controls (green, downregulated; black, same; red, upregulated). Dot plots on the right of the expression heatmap depict experimental classifications of treated specimens based on route of exposure (left to right: intraperitoneal injection, or oral gavage) and nutritional status of vehicle (left to right: not nutritive solution, or nutritive oil-based vehicle). **(C)** Average Log2FC expression ± s.e.m. based on Profiler DEGREEs, split into co-expression patterns from **(B)**, and separated by experimental MOA classification. **(D)** Concordance of relative expression estimates normalized to house-keeping glyceraldehyde-3-phosphate dehydrogenase (Gapdh) gene between RNA-seq and qPCR experiments for eight annotated gene symbols across hepatotoxic agents with DNA damage MOA; all assays were performed from cDNA templates derived using the same total RNA extracts for both techniques (*N* = 3 per hepatotoxic agent); qPCR assays were performed in technical duplicates per reaction plate with matched untreated samples as controls (CTR). Overall linear regression (regression mean: solid black line; ±95 CI of regression: dashed black lines) corresponds to qPCR-based average normalized Log2FC expression levels of each gene (x-axis) vs. normalized Log2FC expression measurements per sample among mRNA transcripts with matching gene symbols detected by RNA-seq (y-axis). **(E)** Enriched signaling pathways and **(F)** inferred upstream regulators for Profiler DEGREEs with |Log2FC|>0.82 under each MOA cluster identified in **(B)**, based on Ingenuity Knowledge Base ontologies; equal analyses are depicted in regards to **(G)** metabolic pathways and **(H)** inferred disease and biological functions, respectively. Pathways depicted in **(E,G)** heatmaps showed enriched representation *p* < 0.05 in at least one MOA cluster; upstream regulators and functions in **(F,H)** showed both enriched representation p < 0.05 and predictive |z| > 2.0 in at least one MOA cluster. Intensity of purple coloring in pathway heatmaps **(E,G)** represent increasing levels of significance; coloring of activation heatmaps **(F,H)** on a blue-white-orange gradient scale depicts prediction z-score values (blue, inhibited; white, inactive; orange, activated). **(I)** Expression heatmap for two-way unsupervised clustering of Log2FC differences based on the 8 biomarkers highlighted in **(B)** (columns). Coloring of the row dendrogram and its labels (right) matches the 7 MOA clusters (I-VII) depicted in **(B)**. Top right: heatmap is colored on a green-black-red gradient scale (top left) of Log2FC values relative to mock-treated controls (green, downregulated; black, same; red: upregulated). Dot plots (left) depict experimental classifications of treated specimens based means of transcriptional activation of MIEs (left to right: non-receptor mediated, or NRM vs. receptor-mediated or RM). *N* = 9 independent specimens per MOA classification with three different hepatotoxic agents each (*N* = 3 per MOA × Agent combination): Ahr (3ME = 3-methylcholantrene, 300 mg/kg/day, 5 days; NAP = β-naphthoflavone, 1,500 mg/kg/day, 5 days; LEF = leflunomide, 60 mg/kg/day, 5 days); CAR/PXR (ECO = econazole, 334 mg/kg/day, 5 days; MET = methimazole, 100 mg/kg/day, 3 days; PHE = phenobarbital, 54 mg/kg/day, 5 days); Cytotoxic (CAR = carbon tetrachloride, 1,175 mg/kg/day, 7 days; CHL = chloroform, 600 mg/kg/day, 5 days; THI = thioacetamide, 200 mg/kg/day, 5 days); DNA damage (AFL = aflatoxin B1, 0.3 mg/kg/day, 5 days; IFO = ifosfamide, 143 mg/kg/day, 3 days; NIT = *N*-nitrosodimethylamine, 10 mg/kg/day, 5 days); and PPARA (BEZ = bezafibrate, 617 mg/kg/day, 7 days; NAF = nafenopin, 338 mg/kg/day, 5 days; PIR = pirinixic acid, 364 mg/kg/day, 5 days). Log2FC measurements were calculated relative to 9 vehicle-only mock-treated controls. The TGxSEQC training dataset comprises 54 total individual specimens with reads aligned onto 30,852 RefSeq-annotated transcripts overall. To designate the final list of Profiler DEGREEs, Log2FC of differentially expressed transcripts (resolution-weighed ANOVA FDR p < 0.05 and δ_Log2FC_ > 0.3 × σ_SSR_, and both *post*-*hoc p* < 0.05 and Log2FC > 95%TI_SSR_ in at least one MOA vs. average of controls) annotated as mRNA-encoding (RefSeq NM_ suffix) were consolidated by the average of Log2FC values under matching and nonduplicate gene symbols from the Entrez database. Reference genome: rn6.

We asked to what extent the DEGREEs detected by our method captured the underlying organization of specimens into MOA groups. Using two-way unsupervised hierarchical clustering (Ward method), we found the set of 1,510 DEGREEs sorted not only 43 of the 45 treated specimens into their original MOA and chemical agent groupings, but also recovered their stratification by route of exposure, nutritional status of vehicle, and one of two means of pathway activation: receptor mediated (RM) and non-receptor mediated (NRM). We also detected five co-expression patterns among DEGREEs, which were supported by separate clustering of correlation scores for the differential expression of DEGREEs across MOA groupings (Figure 4A). Still, we found that clustering based on 1,510 DEGREEs resulted in interleaved MOA groups, except for PPARA (Figure 4A). This kind of transcriptional cross-talk among 1,510 DEGREEs has been previously reported for this same dataset, suggesting that downstream pathways elicited by different chemical agents under the same MOA classification can converge to the same regulatory hubs through different molecular cascades and lead to distinctive transcriptional effects; conversely, chemical agents classified under different MOA can exhibit similar transcriptional signatures just as well (Funderburk et al., 2017).

Next, we investigated the capacity of Profiler DEGREEs to segregate MOA groups, and their underlying strata, compared to the pool of DEGREEs. Unsupervised clustering based on the 65 Profiler DEGREEs revealed seven MOA clades among treated specimens (I-VII) and, like its larger counterpart with 1,510 DEGREEs, five minimal gene co-expression patterns (a–e) (Figure 4B). Using only Profiler DEGREEs also recovered the experimental stratification of the treated specimens with the exception of 6 specimens interspersed between the Cytotoxic, DNA damage, and CAR/PXR MOA groups (Figure 4B). Yet, hierarchical clustering by Profiler DEGREEs also segregated each of the MOA from each other, unlike clustering with the entire pool of 1,510 DEGREEs (Figure 4B). This highly discriminative capacity indicates that Profiler DEGREEs represent the most discriminative subset of DEGs to extract transcriptional signatures.

The ability to segregate entire MOA groups from each other based on Profiler DEGREEs allowed comparing transcriptional profiles of each MOA across the five expression patterns. Among RM MOA groups, PPARA showed the strongest and most dissimilar transcriptional response; it was also the MOA with the most pronounced effects across the entire dataset and across all co-expression patterns (Figure 4C). Both Ahr and CAR/PXR exhibited modest Log2FC levels relative to the effects elicited in the PPARA groups; interestingly, expression regulation trends in Ahr groups were opposite to those in PPARA across all five expression patterns (a–e), and in three for CAR/PXR groups (a-c). Of the three RM groups, CAR/PXR exhibited the weakest differential expression levels. In general, MOA groups with NRM activation show muted expression effects compared to RM MOAs, in both cases reminiscent of CAR/PXR expression trends, and with DNA damage agents showing slightly greater effects than cytotoxic ones (Figure 4C). Even then, LSTNR analysis produced accurate estimates of weak differential expression levels, such as those elicited by DNA damage MOA agents, that showed significant concordance with matching qPCR validation data (Gong et al., 2014) as shown in Figure 4D.

To infer the biological mechanisms underlying the transcriptional signatures detected by unsupervised clustering, we performed pathway enrichment analysis using Profiler DEGREEs for each of the seven MOA clades separately (I-VII; Figure 4B) via the Ingenuity® platform. Profiler DEGREEs in each MOA clade were filtered for inclusion against a threshold |Log2FC|>0.82 (i.e., >1.76 expression fold-changes vs. average of controls). This threshold is the LSTNR filter, and equals the 95% tolerance interval for SNR = 1 among SGs based on residuals of gene Log2FC means within MOA × Agent groups. Expectably, PPARα activation was among the top enriched pathways, indicative not only of the strength of differential expression with PPARA MOA agents, but also of the signature in the AHR and CXR/PXR groups with the same genes, but with opposite expression regulation. Viewed together, the top enriched signaling pathways were consistent with acute-phase inflammation-related mechanisms (e.g., IL1, IL12, complement system), transcriptional regulation of xenobiotic and steroidal pathways (activation of RXR, LXR, FXR, TR, and estrogen-dependent pathways), and mitochondrion-linked metabolic remodeling (AMPK and Ca^2+^ signaling) (Figure 4E).

The results for signaling pathway enrichment analysis were further supported by sets of predicted upstream regulators via activation z-scores (Figure 4F). In particular, from the perspective of the PPARA MOA group, exclusively represented by Clade VII, the strongest predicted regulator was PPARA itself, and was also one among three other inferred activated factors characteristic of PPAR signaling (PPARG, PPARD, and PPARGC1A). Other factors included KLF15 and PML, showing opposite activation scores from each other, and both of which modulate TP53 activation (also predicted) by regulating the activity of lysine acetyltransferases such as EP300 and KAT6A in opposite manners (Haldar et al., 2010; Rokudai et al., 2013). Interestingly, our analysis also inferred the activation of TCF7L2, a transcription factor known to associate with genomic enhancers coincident with epigenetic H3K27ac post-translational modifications (Frietze et al., 2012). Activation of TFAM, the master transcription factor of mtDNA (Ekstrand et al., 2004) and one of the upregulated Profiler DEGREEs in PPARA MOA specimens based on our analysis (Figure 4B), was also predicted by the transcriptional signatures of the rest of the Profiler DEGREEs (Figure 4F).

Inferred regulation scores for upstream regulators were opposite to those in PPARA specimens for all other clades, except for clade IV. The distinct behavior of clade IV, which included two of the three liver specimens treated with carbon tetrachloride, did present distinctive enrichment of LXR/RXR activation and atherosclerosis signaling pathways, along with inferred activation of PPARA and KLF15. Those results for clade IV were consistent with known effects of carbon tetrachloride, a traditional model of chronic liver injury that elicits fibrogenic activity in hepatic stellate cells and loss of fenestration along liver sinusoids due to thickening of basal membranes with fibrillar collagens; both these responses are relayed via LXR signaling (Beaven et al., 2011; Xing et al., 2016). In this context, activation of PPAR signaling would offer a counteracting mechanism to deactivate fibrogenic stellate cells; in fact, PPARA is a known transcriptional regulator with reported protective roles against steatosis in hepatocytes (Tsuchida and Friedman, 2017).

When inspected from a metabolic standpoint, pathway enrichment analysis pointed to three pillars of mitochondrial function: fatty acid β-oxidation, degradation of xenobiotic agents, and sourcing of TCA cycle intermediates via amino acid catabolism (Figure 4G). The same predictions were inferred based on the activation z-scores of biological functions in the Ingenuity Knowledge Base (Figure 4H). Clade IV was the least enriched for pathways involving lipogenic activity, perhaps indicative of stalled fatty acid synthesis in steatotic hepatocytes (Ogrodnik et al., 2017). Once again, clade VII (i.e., PPARA MOA group) presented the most overt levels of significance and predictive inference, and predicted the opposite biological response with respect to all other clades, except for clade V (Figure 4H).

Of note, clade V consists of two agents from different MOA groups: thioacetamide (THI) from the cytotoxic MOA group, and *N*-nitrosodimethylamine (NIT) from the DNA damage group (Figure 4B). Still, the ability of THI to elicit secondary oxidative DNA damage has long been reported in its function as a free-radical generator (Clawson et al., 1997). Enrichment analysis and predicted biological activities suggested that both agents under clade V elicited increased biosynthesis of ketogenic precursors, oxidation of long-chain fatty acids, defective glucose metabolism, and hepatic steatosis—all of which are processes that synergize with mitochondrial respiration and rely on the integrity of the mitochondrial genome. We interpreted the combination of predicted glucose disorders and enhanced metabolism of ketogenic intermediates triggered by exposure to clade V agents to have a compensatory function in response to mtDNA damage. In a sense, these endogenous responses to NIT and THI exposure would be analogous to the effects of ketogenic diets in the Deletor mouse model, in which high-fat feeds ameliorate the chronic bioenergetic crisis that stems from defective maintenance of mtDNA integrity and copy number (Ahola-Erkkilä et al., 2010).

Last, we performed sequential tree partitioning analysis of Profiler DEGREEs to extract candidate biomarkers and test their ability to discriminate MOA groups compared to using all 65 Profiler DEGREEs. The process of biomarker selection was a two-tier process: first, tree partitioning analysis was performed with all MOA groups; then, to preempt disparately larger transcriptional responses in PPARA MOA groups from undercutting our analysis, we repeated the partitioning analysis without including PPARA specimens. We detected eight biomarkers from both analyses combined: Ucp3, Acaa1b, Acaa1a, Sugct, Hadhb, Tfam, Gsdmd, and Tmem86b (Figure 4I). Altogether, these eight biomarkers represented four out of the five co-expression patterns we identified by clustering all 65 Profiler DEGREEs (Figure 4B). The ability to sort treated specimens by biomarker-based clustering into MOA clades was commensurate to that of Profiler DEGREEs with one notable exception, clade V, split into two well defined biomarker-based groups: the first one, for NIT-treated specimens, showed upregulated Gsdmd and downregulated Tmem86b vs. controls; the second one, for THI-treated specimens, showed upregulation of Tfam and downregulation of Sugct instead (Figure 4I). Among the eight biomarkers, Ucp3 showed the most striking differences among MOAs, and clearly distinguished three types of transcriptional responses. In the case of the RM Ahr and CAR/PXR MOAs, the response was consistent with silencing of Ucp3 expression; instead, RM activation of PPARA MOAs exhibited the largest levels of Ucp3 overexpression throughout; finally, NRM activation of cytotoxic and DNA damage pathways were met by modest upregulation of Ucp3 (Figure 4I).

In principle, the purpose of defining a minimal set of biomarkers by subsequent refinements of candidate gene signatures—e.g., DEGREEs down to Profiler DEGREEs, and Profiler DEGREEs down to biomarkers—merely seeks to determine a realistic and manageable set of testable target genes for an experimentalist to carry out validation and reproducibility assays with at the bench. The underlying assumption is that the reductive process involved in curating among candidate targets will not only reflect similar or improved sample partitioning for the selected gene subset vs. the original list of candidates (as shown in Figure 4I), but that the predictive strength of each selected gene improves as the list of genes becomes smaller with higher statistical stringency. To test this, we performed bootstrap forest models using all 1,510 DEGREEs, only the 65 Profiler DEGREEs, or only the 8 biomarkers as candidate lists vs. the inferred MOA clusters (I-VII) depicted in Figure 4B; then, we tracked the predictive strength of the 8 selected biomarker genes common to all lists in terms of *G*2—the likelihood-ratio test—which: (a) represents the relative contribution of a gene among all others to assigning samples into expected clusters across bootstrapped partition trees; and (b) approximates a χ2 distribution to estimate the statistical significance of each gene's predictive capacity. As we surmised, the contributions of biomarker genes to MOA cluster assignment showed increasing significance as the tested candidate gene lists were curated in favor of genes with more robust expression differences (Table 5). Notably, Ucp3 ranked as the top contributor among any candidate genes retained in all tiers of statistical stringency (Table 5). This last observation agreed with our earlier interpretation (based on unsupervised clustering of samples using 1,510 DEGREEs, 65 Profiler DEGREEs, or 8 biomarkers) that Ucp3 overwhelmingly outpaced any other individual gene in the training dataset in its ability to assign samples to their inferred MOA cluster memberships (Figure 4I).

**Table 5:** Partitioning contribution statistics by likelihood ratio tests of eight selected biomarkers via bootstrap forest modeling of sample classification under seven inferred MOA clusters with reference gene lists at different tiers of statistical stringency (mode-of-action training RNAseq dataset, TGxSEQC; GEO accession number: GSE55347).

**Gene Symbol**	**1,510 DEGREEs**	**65 Profiler DEGREEs**	**8 Biomarkers**	**Statistic**
Ucp3	8.4178	14.2830	14.5505	*G2*
	(9.89%)	(17.12%)	(22.92%)	*(G2_*gene*_/G2_*total*_, %)*
	1	1	1	Rank by *G2* [highest: 1]
	0.2091	0.0266	0.0241	*p-*value [*df*: MOA Clusters-1]
Tmem86b	0.0000	2.4276	8.5938	
	(0.00%)	(2.91%)	(13.54%)	
	1363	6	2	
	1.0000	0.8765	0.1977	
Hadhb	0.3465	1.4197	8.3181	
	(0.41%)	(1.70%)	(13.11%)	
	83	20	3	
	0.9992	0.9647	0.2157	
Gsdmd	0.0000	0.1950	7.5273	
	(0.00%)	(0.23%)	(11.86%)	
	603	50	4	
	1.0000	0.9999	0.2748	
Acaa1b	0.0000	2.3052	7.3439	
	(0.00%)	(2.76%)	(11.57%)	
	142	9	5	
	1.0000	0.8896	0.2902	
Acaa1a	0.4146	2.1847	6.5803	
	(0.49%)	(2.62%)	(10.37%)	
	61	12	6	
	0.9987	0.9020	0.3614	
Tfam	0.0000	2.2218	6.3974	
	(0.00%)	(2.66%)	(10.08%)	
	1331	10	7	
	1.0000	0.8982	0.3802	
Sugct	0.0000	0.0000	4.1599	
	(0.00%)	(0.00%)	(6.55%)	
	1308	56	8	
	1.0000	1.0000	0.6551	

Differentially regulated Ucp3 expression across MOAs is particularly relevant in the context of mitochondrial metabolism. Besides functioning as an uncoupler of oxidative phosphorylation, Ucp3 also facilitates fatty acid metabolism and ROS regulation in mitochondria (MacLellan et al., 2005), perhaps through a role for fatty acids as competent ROS scavengers (Lemke et al., 2014). We interpreted the stark contrasts between MOA groups on the basis of Ucp3 expression alone to be indicative of remodeled lipid store management in cells, perhaps to alleviate insults on mtDNA integrity or ROS imbalances. This interpretation is supported by the functions of all other biomarkers detected by the LSTNR method (Figure 4I): (a) Sugct, an enzyme involved in lysine degradation that metabolizes glutarate (Marlaire et al., 2014); (b) Acaa1a and Acaa1b, both enzymes that participate in lipid metabolism in peroxisomal compartments (Schram et al., 1987; Ferdinandusse et al., 2001); (c) Hadhb, a critical subunit of the mitochondrial trifunctional protein that governs fatty acid beta-oxidation inside mitochondria (Spiekerkoetter et al., 2004); (d) the gene encoding for lysoplasmalogenase, Tmem86b (Braverman and Moser, 2012); (e) Gsdmd, or gasdermin D, a lipid-porating protein that effects pyroptotic cell death during inflammation (Rathkey et al., 2017); and (f) Tfam, the master transcription factor for mtDNA (Woo et al., 2012; Stiles et al., 2016).

From a biological perspective, implementation of the LSTNR method unveiled different levels of transcriptional cross-talk in response to chemical exposure. Perhaps unsurprisingly, the ability to discriminate different forms of liver toxicity by transcriptional profiling was founded on maintenance of mitochondrial function, in particular by remodeling lipid metabolism to counterbalance toxic effects on aerobic respiration machinery. Still, the LSTNR method did harbor one particular strength: it provided a systematic strategy to distinguish between interconnected transcriptional signatures and characteristic ones based on tiers of statistical stringency. In that regard, the different tiers of differential gene expression that LSTNR dissects may outline the difference between targeted (or causal) triggers and their secondary (or consequential) effects in transcriptional responses to toxicants. We believe these types of results from LSTNR implementation, as shown by the analysis of the TGxSEQC dataset, reflect the underlying basis of bottom-up transcriptional networks that become more intertwined as more gene nodes are added—and yet, the LSTNR method can unravel them systematically from the top down.

## Discussion

### Independent filtering in LSTNR: data worth making is not always worth keeping

A key element of RNAseq analysis is choosing a sensible threshold that reflects the dynamic range of gene detection based on total aligned reads from a sequencing run. In our analyses of the TCGA and TGxSEQC datasets, we used the scale parameter α of a 3-parameter Weibull fit to gene RPM means as the independent filtering threshold. Mathematically, this ensured no more than 63% of genes with aligned reads will be retained for analysis, since by definition the α parameter is the 37th percentile of a Weibull-distributed variable (Aitkin and Clayton, 1980). In contrast, we found that our approach to independent filtering, based on a lognormal distribution, estimated a dynamic range that was valid for every pseudogene in the EPIG-seq *in silico* dataset. This outcome, which rarely occurs in the analysis of *bona fide* experimental data sets, is consistent with using simulated data since: (a) pseudogene averages in statistical groups are prescribed under known patterns for all pseudoreplicates; and (b) read count variation is modeled around those fixed averages across the entire dataset. Given these properties of simulated data, it follows that an explanatory parametric fit of pseudogene averages should include every pseudogene if those gene averages are also parametrically tailored; if true, the estimated dynamic range of average RPM-values should be valid for all pseudogenes and, consequently, all pseudogenes should pass independent filtering—just as we saw in the EPIG-seq *in silico* dataset.

It is worth mentioning that the point of independent filtering has little to do with estimates of relative expression. In fact, fold-change differences across groups are scored within genes in RNAseq, meaning differential expression measurements for individual genes are separate from each other whether they fall within the dynamic range of detection or not. However, subjecting all genes to multivariate differential expression analyses, including underresolved ones indistinguishable from instrumental noise, undermines the ability to adjust significance tests for multiple comparisons (Benjamini and Hochberg, 1995; Tamhane and Dunlop, 2000). Therefore, if DEGs are selected based on significance scores alone, their numbers will be inflated (Type I error) if underresolved genes are not discarded ahead of inferential testing.

### Empirical fitting of gene-wise coverage

It is in this context that effect size filtering at the gene level becomes particularly relevant to the analysis of deeply sequenced RNAseq data sets. Depending on the type of experimental design, additional levels of DEG discrimination may be needed, for example, when dealing with transient expression differences, such as mtDNA depletion time courses in DN-POLG cells (Martínez-Reyes et al., 2016). In time course experiments, differences in gene expression are expectably smaller between successive time points than comparisons between start and end points. With shorter sampling intervals in an experiment come smaller expression differences—yet instrumental noise, which is relatively fixed, may be more prominent than the dispersion of differential expression measurements. This is one major reason why relying exclusively on *pairwise* significance tests to detect DEGs across 3+ groups are prone to Type II errors. If a practical effect size criterion is not imposed to discriminate genes that show statistical significance (or not) in RNAseq tests with small sample sizes, it is difficult to separate between genes whose significant expression differences are more likely to be real, rather than anecdotal, in an underpowered experimental design (Ioannidis, 2005).

Average sequencing depth across detected genes in RNAseq experiments account predominantly for random dispersion and instrumental error combined—except in DEGs. In that sense, one can interpret the behavior of accrued reads in each detected gene as a fingerprint of how read counts vary with sequencing depth. From such perspective, the within-gene sample size is the total number of experimental samples, and the between genes sample size is the number of genes retained after independent filtering. One can then “studentize” read count variation across gene × group statistical blocks by producing an “image” of transformed RPM rates. This can be achieved by fitting a generalized linear model (GLM), which are statistical instruments to account for magnitude and resolution differences all at once in non-linear and non-Gaussian systems (Nelder and Wedderburn, 1972).

To perform GLM, it is necessary to restate an observed probability density function in the general form of the exponential family. The purpose is to devise a transformation that turns RPM data into a set of values showing a linear relationship between samples and their averages, known as a *linear predictor* or transformant ***B***(**ϑ**) [or ***B*** in short]; furthermore, if the chosen linear predictor ***B*** yields transformant values whose variation around within-gene averages behave like a classical distribution from the exponential family (e.g., normal, binomial, Poisson, or exponential), then the transformant values of ***B*** can be manipulated algebraically into normally distributed scores using what is known as a *link function* **η**(***B***) [or **η** in short]. As a result, **η** is a representation of RPM that, unlike RPM-values themselves, are linear and normally distributed—which meets requirements for ordinary multivariate ANOVA tests of significance; this is the basis of GLM (see Supplementary Methods) (Nelder and Wedderburn, 1972).

The metric of interest when using GLM to model gene-wise RPM averages is not the actual values of the transformant ***B***-values, but what those values represent: an instrument to explain which genes are *better* resolved based on statistical evidence about their location in the dynamic range of sequenced read detection. In that context, significance levels of genes based on ANOVA testing of the linear predictor ***B*** are a proxy for how robust are the differences between groups within one gene vs. all other genes based on their RPM data and its non-linear variation. Therefore, those significance scores rank the experiment's sensitivity to changes in each gene. In the LSTNR method, we used these scores to produce gene “resolution weights” when testing log-fold expression measurements.

The weight function of choice is the cumulative hazard of gene significance scores. The cumulative hazard function, which is the complement of the cumulative density function in negative logarithmic scale, becomes a score for how strong is the expectation that read counts from individual genes estimate “true” relative expression differences between groups. The cumulative hazard function has many advantages in regards to how it represents RPM resolution: it can be determined from bounded population data (i.e., the number of detected genes is a finite number), it is continuous-valued (i.e., not ordinal), and monotonically increasing (larger weights for better resolved genes). Also, it is derived from read count data without being a direct transformation of read count values; the benefit is that the same weight can be applied to each gene across samples or experimental realizations whether net read counts are the same or not between individual replicates. In the context of RNAseq output, the behavior of cumulative hazard functions resembles that of the “tightness” of relative expression measurements around the mean from sequencing data: genes with low read counts are poorly detected and yield noisy estimates of log-fold differences; genes with more aligned reads produce more robust estimates with increasing coverage; and genes near saturated sequencing levels plateau to the absolute resolution limit of the sequencing instrument.

Based on the TCGA dataset, we found the behavior of resolution weights to be consistent with how read count differences in RNAseq data behave: genes with better resolution will often present either low read counts and large differences between groups, or high read counts with modest differences and tight within-group variability. Since RNAseq is based on PCR amplification, observing tight distributions for abundant transcripts may occur rarely, since amplification errors are propagated exponentially. Using separate implementations of the LSTNR method in each individual realization ameliorated the impact of such features, all too common in noisy RNAseq data. In effect, resolution scaling in the LSTNR method adjusted for differential expression tests for exponential propagation of PCR errors.

In theory, there is an additional benefit worth noting that LSTNR offers. GLM with canonical link functions relies on the statistical sufficiency of exponential family distributions, and the resolution weights of genes are derived from linear predictor ANOVA based on ranks, not scores, of statistical significance. If individual experimental replicates are a true representative sample of a population, and their distribution belongs to the exponential family (as implemented in the LSTNR method), then a notable statistical corollary follows: the resolution power of gene detection from one experiment is valid for separate realizations from the same population. In practical terms, this means the resolution weights for genes detected in one study are valid for any additional sequencing of its replicates, entire repeated experiments, and replicate studies—*regardless of sample size or sequencing depth*. If true, the GLM basis of the LSTNR method makes it a particularly attractive pipeline to determine consensus resolution weight matrices from small training sets heading into consolidated meta-analyses of much larger cohorts. As our findings suggests, the use of LSTNR-derived gene weight matrices would protect DEG analysis against detrimental batch effects and subsampling errors, both of which are characteristic of epidemiological and clinical studies.

### Detecting DEGS by resolution-weighed multivariate analysis in the LSTNR method

The customary methods of inferential testing among multiple groups are ANOVA models because of their computational efficiency and ease of implementation. The main caveat of ANOVA models pertains statistical power, which strongly depends on sample sizes, normally distributed responses, and homoscedasticity across statistical blocks. These requirements are a major impediment to the management of RNAseq data sets; for one, sample sizes are limiting in RNAseq applications due to cost constraints, since sequencing reagents, equipment and necessary computational support to convert raw sequencing output into genome-aligned reads are all expensive. In regard to normality of measurements, it is common practice to represent expression levels as log-transformed read counts, such that expression fold-changes between groups become arithmetic differences between means with distributions that approximate normality; this is not only questionable for most RNAseq experiments, mainly due to the impedingly small sample sizes mentioned earlier, but also insufficient as it does not address lack of homoscedasticity.

Log2FC values, which are read count ratios, are a measurement of relative expression levels for a gene between conditions; however, Log2FC values by themselves do not offer any information on their own measurement resolution because the read counts used to estimate them are “divided out.” Calculating significance scores based on Log2FC values is only statistically fair if all genes show equal levels of read coverage or, as in the LSTNR method, if relative expression measurements are adjusted to reflect different resolution levels among detected genes. Introducing resolution weights to multivariate ANOVA testing of relative expression differences posited attractive statistical advantages. In principle, resolution scaling of log-fold differences should: (a) improve homoscedasticity across genes, as shown in Figure 2D; (b) discriminate between highly variable (largest fold-differences) and highly granular (largest read counts) genes, as shown in Figures 2C, 3B; and (c) prioritize genes whose prospective differential expression are the most reproducible should RNAseq experiments be repeated. Above all else, the issue of reproducibility described in (c) was the reason behind our split-pool analyses approach to the TCGA dataset, and the identification of a consensus set of Profiler DEGREEs (see Table 3).

When we analyzed the breast cancer dataset from TCGA, we found large discrepancies in the number of significantly resolved genes among separate analyses, yet the number of SGs was very consistent across the board. In our view, this illustrates the main strength behind the LSTNR method: whether particular sample groupings lead to statistically significant dispersion in RPM-values in one subset vs. another is less relevant than the actual ranking of their relative resolution within replicate experiments. Our interpretation is based on the premise that estimates of variability in observed RPM-values within genes are representative of how accurately they were detected *by instrumentation* with respect to each other, not whether accuracy in sequencing was exactly the same for all detected genes between two different replicate experiments. In that sense, our findings showed how coupling of individual sequencing resolution of genes with relative expression differences in the LSTNR method alleviates discrepancies in the number of detectable SGs across replicate experiments, and performs well when the statistical groups are well-defined in advance—e.g., treatments, phenotypes, controls, etc.

### The LSTNR method can estimate signal, noise, and reproducibility benchmarks

Benchmarking the expected dispersion range in RNAseq output is the foundation to a power analysis that outlines what are the smallest differences to expect ahead of deeper sequencing; often, though, access to RNAseq and costs are too prohibitive to pursue any benchmarking efforts. In such cases, the same projection for expected dispersion ranges can be used as a practical signal-to-noise (SNR) threshold of reproducibility that a select subset of genes can be validated against, even if the expression differences measured with RNAseq data for those genes were statistically significant or not. A benchmark SNR threshold of within-gene dispersion in sequenced RPM-values is a key metric for experimentalists, because it helps justify or rule out additional rounds of sequencing (or patient recruitment in clinical settings) when the statistical significance of any transcriptional differences is marginal—or, if all else fails, to shift focus to better experimental alternatives or re-design a project altogether.

To perform relative expression analysis, we determined a reference expression value in each gene equal to the average log_2_(RPM) in a reference control group. The advantage of establishing a fixed “null hypothesis” reference, instead of using the distribution of samples in the control group, is that sample means and variation from individual replicates can be estimated for all groups, including the baseline condition, and before inferential testing of significance. The benefits are many: in practice, RNAseq experiments often produce expression measurements that, in retrospective, are underpowered, biased or unable to detect relevant biological effects because intrinsic biological variability among specimens is simply too large; in other cases, cost constraints limit studies using RNAseq technology to low replication models (Conesa et al., 2016). Faced with these challenges, one could instead project from existing data (even if it is only available for control samples) how large the dispersion in RNAseq output will be; for example, we calculated the predicted 95% tolerance intervals around the mean Log2FC measurements as a surrogate estimate of expectable measurement variability. Those tolerance levels, which can be calculated directly from the residuals of DEGs, represent a transcriptome-wide signal-to-noise (SNR) thresholds of practical reproducibility. Furthermore, since these are based on the variation of individual Log2FC measurements around means of gene × group blocks, they project the scales of expression differences that should be reproduced by other techniques (e.g., targeted qPCR) or in repeated experiments with reasonable sample sizes (e.g., 95% prediction intervals). In the case of the simulated EPIG-seq data, our results suggested that dispersion of Log2FC measurements was roughly the same whether it was calculated based on all genes passing filter or only on statistically significant ones (Figure 2G). This entails that SNR-based criteria for reproducible differential expression can be established ahead of statistical testing based solely on the distribution of Log2FC residuals. In practical terms, would allow confirmatory qPCR assays to be designed from low replication RNAseq studies, simply because noise benchmarking does not require detecting DEGs.

We must point out, going back to the EPIG-seq *in silico* dataset, how the 73 patterned pseudogenes that did not match their simulated trends originated from prescribed *in silico* patterns A, C, or E (Figure 2E). All these three patterns show higher average expression levels in all treatment groups vs. the baseline average; in contrast, the matched patterns B and D both exhibit a dominant downregulation trend: expression in pattern B pseudogenes decreases further when looking across treatment groups, and pattern D exhibits a return to baseline after an “expression spike” in the first treatment group (Figure 2H). These results, which suggest that downregulation trends are easier to discriminate than upregulation ones, may simply reflect the fact that resolution is finite—meaning the number of possible values for RPM differences is countable, since they are ultimately based on integer-based read counts. This implies that, when the number of read counts drops relative to a control, it reduces the number of combinations available to measure gene expression ratios. To clustering algorithms, larger downregulation differences may become easier to discriminate as they behave more like piecewise jumps. In contrast, differences of equal magnitude, but upregulated relative to controls, tend toward a continuum because the scale of possible values is refined with increasing numbers of reads per gene—and, by similar logic, upregulation differences are harder to discriminate by clustering analysis. Such behavior of countable differences in read counts has somewhat intuitive implications: for genes with low coverage to be detected as differentially expressed, both the net and proportional differences in read counts between groups must be large and far from the instrumental background so as to be more accurate and less piecewise; for genes with high read counts, significance is possible for smaller and more precise proportional differences, but is only justifiable if the variation in net read counts is also tight. Above everything else, these are the governing premises behind our design strategy for the LSTNR method.

### Co-expression patterns detected through LSTNR method are reproducible at different statistical stringency levels

To define candidate biomarkers for different transcriptional signatures, the LSTNR method defines a subset of Profiler DEGREEs, equal to the largest subset of ranked DEGREEs with monotonically decreasing retrospective statistical power (≪π≫ >90%) and effect size Δ_Log2FC_ *at the same time*. The rationale behind this approach is to maximize discriminatory potential among transcriptional signatures of each breast cancer specimen by accounting for the largest possible differences between subtypes (i.e., effect size) while minimizing the commensurate false-positive rate projected from the available experimental data (statistical power).

In both experimental datasets, Profiler DEGREEs matched the discriminant capacity of other pipelines, but with considerably less genes overall. The strongest evidence for this came from our analysis of the TGxSEQC training dataset. If stratified by agent, the TGxSEQC dataset is a 15-group experimental design with minimal replication level (*N* = 3). Hence, it cannot be split into realizations for parallel testing, and has to be tested through a single-shot analysis. Another challenge to testing the TGxSEQC training set deals with prospective statistical power: given 15 groups, the number of possible comparisons between them is very large. Therefore, if the analysis is performed based on pairwise gene expression differences, there is a large risk of over-correcting for multiple testing. As a result, this would underestimate the number of DEGs, and limit the ability to discriminate specimens from different MOA groups. Consequently, grouping the pool of 45 specimens under too many categories is detrimental to the statistical power of the experimental design as a whole. To circumvent this challenge, and based on the fact that each of the individual chemical agents belongs to one out of five overarching MOAs, we opted to group specimens under one out of five MOA groups with *N* = 9. Even then, analysis of the TGxSEQC training set using the LSTNR method captured additional sample classifications at different tiers of statistical stringency, based on MOA information alone, and simply by unsupervised hierarchical clustering. In addition, each of the three tiers of discriminant power (i.e., based on 1,510 DEGREEs, 65 Profiler DEGREEs, and 8 biomarkers) were consistent with each other in regards to clustering and detection of co-expression patterns. Most importantly, each increasingly stringent filter improved on the ability of its predecessor to segregate individual MOA groups: clustering by 1,510 separated MOA × Agent groups, but displayed interlaced strata from RM and NRM activation modes; based on 65 Profiler DEGREEs, clustering effectively separated each MOA from all others; and the 8 biomarkers effectively refined the discriminative power between NRM cytotoxic and DNA damage MOA strata.

## Conclusion

We put forth the LSTNR method as an alternative pipeline to tackle current obstacles in RNAseq analysis: first, it defines a detection limit for genes with respect to random errors in instrumental sequencing and read alignment by fitting the observed distribution of aligned read counts; in return, this empirical fit offers a data-driven threshold for independent filtering. Then, the pipeline accounts for non-linear and non-homogeneously distributed variation in read counts per gene; this is used to generate a gene-wise resolution score based on read counts that, when implemented as a weight function, improves the normality and homogeneity of relative expression measurements. With the LSTNR method, the improvements in homoscedasticity by resolution scaling of Log2FC differences allow experimental designs with multiple groups to be tested by standard ordinary ANOVA techniques. Furthermore, DEGs detected by the LSTNR method capture the same transcriptional signatures at different tiers of statistical stringency with high accuracy; we found the performance of the LSTNR method was so robust that it required less genes than previously reported to discriminate breast cancer phenotypes and hepatotoxic MOAs using the same experimental datasets (Perou et al., 2000; Cancer Genome Atlas, 2012; Gong et al., 2014; Wang et al., 2014; Li and Bushel, 2016; Funderburk et al., 2017). As an added benefit, the LSTNR method can produce noise benchmarking estimates to validate RNAseq experiments against by benchtop techniques, regardless of statistical significance or sample replication levels. Altogether, these features set the LSTNR method apart as an agnostic pipeline that: (a) can be programmed for automated processing of RNAseq data with minimal user intervention; and (b) can estimate thresholds of experimental reproducibility for confirmatory assays using RNAseq studies with limiting sample sizes.

## Author contributions

OL conceptualized and implemented the analytical methodology described in the study, performed data analysis, and wrote the manuscript. OL and JS wrote the initial manuscript drafts leading to submission. JS and RW supervised the selection of simulated and experimental RNAseq data sets to validate and test the statistical approach described herein. All authors contributed in the design of the study and interpretation of the results, as well as revised, read, and approved the submitted version.

### Conflict of interest statement

The authors declare that the research was conducted in the absence of any commercial or financial relationships that could be construed as a potential conflict of interest.

## References

[B1] Ahola-Erkkilä S. Carroll C. J. Peltola-Mjösund K. Tulkki V. Mattila I. Seppänen-Laakso T.. (2010). Ketogenic diet slows down mitochondrial myopathy progression in mice. Hum. Mol. Genet. 19, 1974–1984. 10.1093/hmg/ddq076 20167576

[B2] Aird D. Ross M. G. Chen W. S. Danielsson M. Fennell T. Russ C.. (2011). Analyzing and minimizing PCR amplification bias in Illumina sequencing libraries. Genome Biol. 12:R18. 10.1186/gb-2011-12-2-r18 21338519 PMC3188800

[B3] Aitkin M. Clayton D. (1980). The fitting of exponential, weibull and extreme value distributions to complex censored survival data using GLIM. J. R. Stat. Soc. Ser. C 29, 156–163. 10.2307/2986301

[B4] Auer P. L. Doerge R. W. (2010). Statistical design and analysis of RNA sequencing data. Genetics 185, 405–416. 10.1534/genetics.110.114983 20439781 PMC2881125

[B5] Beaven S. W. Wroblewski K. Wang J. Hong C. Bensinger S. Tsukamoto H.. (2011). Liver X receptor signaling is a determinant of stellate cell activation and susceptibility to fibrotic liver disease. Gastroenterology 140, 1052–1062. 10.1053/j.gastro.2010.11.053 21134374 PMC3049833

[B6] Benjamini Y. Hochberg Y. (1995). Controlling the false discovery rate: a practical and powerful approach to multiple testing. J. R. Stat. Soc. Ser. B 57, 289–300.

[B7] Benjamini Y. Speed T. P. (2012). Summarizing and correcting the GC content bias in high-throughput sequencing. Nucleic Acids Res. 40:e72. 10.1093/nar/gks001 22323520 PMC3378858

[B8] Braverman N. E. Moser A. B. (2012). Functions of plasmalogen lipids in health and disease. Biochim. Biophys. Acta 1822, 1442–1452. 10.1016/j.bbadis.2012.05.008 22627108

[B9] Bullard J. H. Purdom E. Hansen K. D. Dudoit S. (2010). Evaluation of statistical methods for normalization and differential expression in mRNA-Seq experiments. BMC Bioinformatics 11:94. 10.1186/1471-2105-11-94 20167110 PMC2838869

[B10] Cancer Genome Atlas N. (2012). Comprehensive molecular portraits of human breast tumours. Nature 490, 61–70. 10.1038/nature11412 23000897 PMC3465532

[B11] Chou J. W. Zhou T. Kaufmann W. K. Paules R. S. Bushel P. R. (2007). Extracting gene expression patterns and identifying co-expressed genes from microarray data reveals biologically responsive processes. BMC Bioinformatics 8:427. 10.1186/1471-2105-8-427 17980031 PMC2194742

[B12] Clawson G. A. Benedict C. M. Kelley M. R. Weisz J. (1997). Focal nuclear hepatocyte response to oxidative damage following low dose thioacetamide intoxication. Carcinogenesis 18, 1663–1668. 10.1093/carcin/18.8.1663 9276646

[B13] Cloonan N. Forrest A. R. Kolle G. Gardiner B. B. Faulkner G. J. Brown M. K.. (2008). Stem cell transcriptome profiling via massive-scale mRNA sequencing. Nat. Methods 5, 613–619. 10.1038/nmeth.1223 18516046

[B14] Conesa A. Madrigal P. Tarazona S. Gomez-Cabrero D. Cervera A. McPherson A. (2016). A survey of best practices for RNA-seq data analysis. Genome Biol. 17:13 10.1186/s13059-016-0881-8 26813401 PMC4728800

[B15] Ekstrand M. I. Falkenberg M. Rantanen A. Park C. B. Gaspari M. Hultenby K.. (2004). Mitochondrial transcription factor A regulates mtDNA copy number in mammals. Hum. Mol. Genet. 13, 935–944. 10.1093/hmg/ddh109 15016765

[B16] Ferdinandusse S. Denis S. Mooijer P. A. Zhang Z. Reddy J. K. Spector A. A.. (2001). Identification of the peroxisomal beta-oxidation enzymes involved in the biosynthesis of docosahexaenoic acid. J. Lipid Res. 42, 1987–1995. 11734571

[B17] Finotello F. Di Camillo B. (2015). Measuring differential gene expression with RNA-seq: challenges and strategies for data analysis. Brief. Funct. Genomics 14, 130–142. 10.1093/bfgp/elu035 25240000

[B18] Frietze S. Wang R. Yao L. Tak Y. G. Ye Z. Gaddis M.. (2012). Cell type-specific binding patterns reveal that TCF7L2 can be tethered to the genome by association with GATA3. Genome Biol. 13:R52. 10.1186/gb-2012-13-9-r52 22951069 PMC3491396

[B19] Funderburk K. M. Auerbach S. S. Bushel P. R. (2017). Crosstalk between receptor and non-receptor mediated chemical modes of action in rat livers converges through a dysregulated gene expression network at tumor suppressor Tp53. Front. Genet. 8:157. 10.3389/fgene.2017.00157 29114260 PMC5660693

[B20] Gong B. Wang C. Su Z. Hong H. Thierry-Mieg J. Thierry-Mieg D.. (2014). Transcriptomic profiling of rat liver samples in a comprehensive study design by RNA-Seq. Sci. Data 1:140021. 10.1038/sdata.2014.21 25977778 PMC4322565

[B21] Hahn G. J. Meeker W. Q. (1991). Statistical Intervals: A Guide for Practitioners. New York, NY: Wiley.

[B22] Haldar S. M. Lu Y. Jeyaraj D. Kawanami D. Cui Y. Eapen S. J.. (2010). Klf15 deficiency is a molecular link between heart failure and aortic aneurysm formation. Sci. Transl. Med. 2:26ra26. 10.1126/scitranslmed.3000502 20375365 PMC3003709

[B23] Hansen K. D. Brenner S. E. Dudoit S. (2010). Biases in Illumina transcriptome sequencing caused by random hexamer priming. Nucleic Acids Res. 38:e131. 10.1093/nar/gkq224 20395217 PMC2896536

[B24] Hansen K. D. Wu Z. Irizarry R. A. Leek J. T. (2011). Sequencing technology does not eliminate biological variability. Nat. Biotechnol. 29, 572–573. 10.1038/nbt.1910 21747377 PMC3137276

[B25] Ioannidis J. P. (2005). Why most published research findings are false. PLoS Med. 2:e124. 10.1371/journal.pmed.0020124 16060722 PMC1182327

[B26] Law C. W. Chen Y. Shi W. Smyth G. K. (2014). voom: precision weights unlock linear model analysis tools for RNA-seq read counts. Genome Biol. 15:R29. 10.1186/gb-2014-15-2-r29 24485249 PMC4053721

[B27] Lemke R. A. Peterson A. C. Ziegelhoffer E. C. Westphall M. S. Tjellström H. Coon J. J.. (2014). Synthesis and scavenging role of furan fatty acids. Proc. Natl. Acad. Sci. U.S.A. 111, E3450–E3457. 10.1073/pnas.1405520111 25092314 PMC4143029

[B28] Li J. Bushel P. R. (2016). EPIG-Seq: extracting patterns and identifying co-expressed genes from RNA-Seq data. BMC Genomics 17:255. 10.1186/s12864-016-2584-7 27004791 PMC4804494

[B29] Li J. Tibshirani R. (2013). Finding consistent patterns: a nonparametric approach for identifying differential expression in RNA-Seq data. Stat. Methods Med. Res. 22, 519–536. 10.1177/0962280211428386 22127579 PMC4605138

[B30] Livak K. J. Schmittgen T. D. (2001). Analysis of relative gene expression data using real-time quantitative PCR and the 2^−ΔΔC^T method. Methods 25, 402–408. 10.1006/meth.2001.1262 11846609

[B31] MacLellan J. D. Gerrits M. F. Gowing A. Smith P. J. Wheeler M. B. Harper M. E. (2005). Physiological increases in uncoupling protein 3 augment fatty acid oxidation and decrease reactive oxygen species production without uncoupling respiration in muscle cells. Diabetes 54, 2343–2350. 10.2337/diabetes.54.8.2343 16046300

[B32] Marlaire S. Van Schaftingen E. Veiga-da-Cunha M. (2014). C7orf10 encodes succinate-hydroxymethylglutarate CoA-transferase, the enzyme that converts glutarate to glutaryl-CoA. J. Inherit. Metab. Dis. 37, 13–19. 10.1007/s10545-013-9632-0 23893049

[B33] Martínez-Reyes I. Diebold L. P. Kong H. Schieber M. Huang H. Hensley C. T.. (2016). TCA cycle and mitochondrial membrane potential are necessary for diverse biological functions. Mol. Cell. 61, 199–209. 10.1016/j.molcel.2015.12.002 26725009 PMC4724312

[B34] Mortazavi A. Williams B. A. McCue K. Schaeffer L. Wold B. (2008). Mapping and quantifying mammalian transcriptomes by RNA-Seq. Nat. Methods 5, 621–628. 10.1038/nmeth.1226 18516045 PMC13303166

[B35] Nazarov P. V. Muller A. Kaoma T. Nicot N. Maximo C. Birembaut P.. (2017). RNA sequencing and transcriptome arrays analyses show opposing results for alternative splicing in patient derived samples. BMC Genomics 18:443. 10.1186/s12864-017-3819-y 28587590 PMC5461714

[B36] Nelder J. A. Wedderburn R. W. (1972). Generalized linear models. J. R. Stat. Soc. Ser. A-G 135, 370–384.

[B37] Oberg A. L. Bot B. M. Grill D. E. Poland G. A. Therneau T. M. (2012). Technical and biological variance structure in mRNA-Seq data: life in the real world. BMC Genomics 13:304. 10.1186/1471-2164-13-304 22769017 PMC3505161

[B38] Odeh R. E. Owen D. B. (1980). Tables for Normal Tolerance Limits, Sampling Plans, and Screening. New York, NY: Dekker.

[B39] Ogrodnik M. Miwa S. Tchkonia T. Tiniakos D. Wilson C. L. Lahat A.. (2017). Cellular senescence drives age-dependent hepatic steatosis. Nat. Commun. 8:15691. 10.1038/ncomms15691 28608850 PMC5474745

[B40] Oshlack A. Robinson M. D. Young M. D. (2010). From RNA-seq reads to differential expression results. Genome Biol. 11:220. 10.1186/gb-2010-11-12-220 21176179 PMC3046478

[B41] Parker J. S. Mullins M. Cheang M. C. Leung S. Voduc D. Vickery T.. (2009). Supervised risk predictor of breast cancer based on intrinsic subtypes. J. Clin. Oncol. 27, 1160–1167. 10.1200/JCO.2008.18.1370 19204204 PMC2667820

[B42] Perou C. M. Sørlie T. Eisen M. B. van de Rijn M. Jeffrey S. S. Rees C. A.. (2000). Molecular portraits of human breast tumours. Nature 406, 747–752. 10.1038/35021093 10963602

[B43] Pfaffl M. W. (2001). A new mathematical model for relative quantification in real-time RT-PCR. Nucleic Acids Res. 29:e45. 10.1093/nar/29.9.e45 11328886 PMC55695

[B44] Rathkey J. K. Benson B. L. Chirieleison S. M. Yang J. Xiao T. S. Dubyak G. R.. (2017). Live-cell visualization of gasdermin D-driven pyroptotic cell death. J. Biol. Chem. 292, 14649–14658. 10.1074/jbc.M117.797217 28726636 PMC5582855

[B45] Risso D. Schwartz K. Sherlock G. Dudoit S. (2011). GC-content normalization for RNA-Seq data. BMC Bioinformatics 12:480. 10.1186/1471-2105-12-480 22177264 PMC3315510

[B46] Robles J. A. Qureshi S. E. Stephen S. J. Wilson S. R. Burden C. J. Taylor J. M. (2012). Efficient experimental design and analysis strategies for the detection of differential expression using RNA-Sequencing. BMC Genomics 13:484. 10.1186/1471-2164-13-484 22985019 PMC3560154

[B47] Rokudai S. Laptenko O. Arnal S. M. Taya Y. Kitabayashi I. Prives C. (2013). MOZ increases p53 acetylation and premature senescence through its complex formation with PML. Proc. Natl. Acad. Sci. U.S.A. 110, 3895–3900. 10.1073/pnas.1300490110 23431171 PMC3593914

[B48] Roy N. C. Altermann E. Park Z. A. McNabb W. C. (2011). A comparison of analog and Next-Generation transcriptomic tools for mammalian studies. Brief. Funct. Genomics 10, 135–150. 10.1093/bfgp/elr005 21389008

[B49] Schram A. W. Goldfischer S. van Roermund C. W. Brouwer-Kelder E. M. Collins J. Hashimoto T.. (1987). Human peroxisomal 3-oxoacyl-coenzyme A thiolase deficiency. Proc. Natl. Acad. Sci. U.S.A. 84, 2494–2496. 10.1073/pnas.84.8.2494 2882519 PMC304678

[B50] Sørlie T. Perou C. M. Tibshirani R. Aas T. Geisler S. Johnsen H.. (2001). Gene expression patterns of breast carcinomas distinguish tumor subclasses with clinical implications. Proc. Natl. Acad. Sci. U.S.A. 98, 10869–10874. 10.1073/pnas.191367098 11553815 PMC58566

[B51] Spiekerkoetter U. Khuchua Z. Yue Z. Bennett M. J. Strauss A. W. (2004). General mitochondrial trifunctional protein (TFP) deficiency as a result of either alpha- or beta-subunit mutations exhibits similar phenotypes because mutations in either subunit alter TFP complex expression and subunit turnover. Pediatr. Res. 55, 190–196. 10.1203/01.PDR.0000103931.80055.06 14630990

[B52] Stiles A. R. Simon M. T. Stover A. Eftekharian S. Khanlou N. Wang H. L.. (2016). Mutations in TFAM, encoding mitochondrial transcription factor A, cause neonatal liver failure associated with mtDNA depletion. Mol. Genet. Metab. 119, 91–99. 10.1016/j.ymgme.2016.07.001 27448789

[B53] Tamhane A. C. Dunlop D. D. (2000). Statistics and Data Analysis: From Elementary to Intermediate (Upper Saddle River, NJ: Prentice Hall).

[B54] Tarazona S. Garcia-Alcalde F. Dopazo J. Ferrer A. Conesa A. (2011). Differential expression in RNA-seq: a matter of depth. Genome Res. 21, 2213–2223. 10.1101/gr.124321.111 21903743 PMC3227109

[B55] Tsuchida T. Friedman S. L. (2017). Mechanisms of hepatic stellate cell activation. Nat. Rev. Gastroenterol. Hepatol. 14, 397–411. 10.1038/nrgastro.2017.38 28487545

[B56] Wang C. Gong B. Bushel P. R. Thierry-Mieg J. Thierry-Mieg D. Xu J.. (2014). The concordance between RNA-seq and microarray data depends on chemical treatment and transcript abundance. Nat. Biotechnol. 32, 926–932. 10.1038/nbt.3001 25150839 PMC4243706

[B57] Woo D. K. Green P. D. Santos J. H. D'Souza A. D. Walther Z. Martin W. D.. (2012). Mitochondrial genome instability and ROS enhance intestinal tumorigenesis in APC(Min/+) mice. Am. J. Pathol. 180, 24–31. 10.1016/j.ajpath.2011.10.003 22056359 PMC3338350

[B58] Wu H. Wang C. Wu Z. (2013). A new shrinkage estimator for dispersion improves differential expression detection in RNA-seq data. Biostatistics 14, 232–243. 10.1093/biostatistics/kxs033 23001152 PMC3590927

[B59] Xing Y. Zhao T. Gao X. Wu Y. (2016). Liver X receptor alpha is essential for the capillarization of liver sinusoidal endothelial cells in liver injury. Sci. Rep. 6:21309. 10.1038/srep21309 26887957 PMC4758044

