# Supplementary material for: A Leveraged Signal-to-Noise Ratio (LSTNR) Method to Extract Differentially Expressed Genes and Multivariate Patterns of Expression From Noisy and Low-Replication RNAseq Data
Source: Front Genet. 2018 May 16;9:176. doi: 10.3389/fgene.2018.00176 (PMC5964166; doi:10.3389/fgene.2018.00176)
Supplement: Supplementary file 3 [file Data_Sheet_1.pdf]

*Supplementary Material*

# A Leveraged Signal-To-Noise Ratio (LSTNR) Method to Extract Differentially Expressed Genes and Multivariate Patterns of Expression From Noisy and Low-Replication RNAseq Data

**Oswaldo A. Lozoya[1]*, Janine H. Santos[1], Richard P. Woychik[1]***

[1]Genome Integrity and Structural Biology Laboratory, National Institute of Environmental Health Sciences, National Institutes of Health, Research Triangle Park, NC, USA

***Correspondence:**
Oswaldo Alonso Lozoya, Ph.D.
oswaldo.lozoya@nih.gov

# 1 Supplementary Methods

## 1.1 *In silico* simulated dataset

An *in silico* dataset of simulated RNAseq counts was used to validate the performance of the LSTNR method. The simulated data set was originally assembled in the development of EPIG-seq, a similarity scoring methodology for count-based data that catalogs co-expressed genes under patterns of differential expression among multiple conditions (1). In short, the EPIG-seq simulated data set comprises 20,000-pseudogene profiles from four equally sized statistical groups (one baseline and three treatments, with N=35 each) that exhibit five prescribed patterns of differential expression. Each of the five co-expression patterns are composed of 200 differentially expressed pseudogenes with respect to the mean expression levels in the baseline group at least in one of the three treatment groups. For genes in each simulated expression pattern, randomly assigned noise to replicates in read counts around the average of their statistical group followed a negative binomial distribution model with a dispersion parameter estimate based on empirical RNAseq data from 10 breast cancer primary tumors deposited in The Cancer Genome Atlas (TCGA) (2). Noise was modeled separately for each expression pattern. Calculated differences between statistical groups within the remaining "unpatterned" subset of 19,000 pseudogenes reflect only randomly assigned noise.

## 1.2 Publicly available breast cancer RNAseq data from TCGA

Count-level RNAseq data from breast ductal carcinoma deposited in TCGA (2) and randomly subsampled was kindly shared by Li and Bushel (1) to assess the performance of the LSTNR v. the EPIG-seq method. Briefly, four independent subsets of breast cancer data were assembled by randomly selecting 10 sequenced lanes each of normal breast tissues (control group) and four breast cancer molecular subtypes (disease groups): luminal A, luminal B, Her2-enriched and basal-like (3-5) among samples in TCGA produced on Illumina GAII sequencers. Further details on the alignment of the RNAseq reads and bioinformatics pipeline are available in the EPIG-seq publication by Li and Bushel (1).

## 1.3 Mode-of-Action Toxicogenomics RNAseq data from the TGxSEQC crowdsource effort

A training count-level RNAseq dataset from the MAQC phase III SEQC crowd source toxicogenomics (TGxSEQC) effort (6, 7) was acquired from livers of male Sprague-Dawley rats after exposure to hepatotoxic agents that share modes of action (MOA). These are defined as the set of biological targets, or molecular initiating events (MIEs), that trigger injury response mechanisms after exposure to a chemical. The TGxSEQC MOA training set is available in the National Center for Biotechnology Information Sequence Read Archive (SRA) (8) under accession number SRP039021 and in Gene Expression Omnibus under accession number GSE55347 . The experimental design included 15 chemicals or vehicle and route-matched controls, with sets of three chemicals sharing one of five MOAs tested under three different exposure routes. Daily dosage was experimentally determined for each chemical agent at the 5-day maximum tolerated dose (MTD), resulting in 5% – 10% body weight reductions v. controls. Animals received a daily MTD of each chemical for 3, 5 or 7 days (depending on the chemical), and single 6-mm punch biopsies from the liver of each animal were harvested 24 hours after the last dose. Three of the five MOAs are associated with well-defined receptor-mediated processes – peroxisome proliferator-activated receptor alpha (PPARA), orphan nuclear hormone receptors (CAR/PXR) and aryl hydrocarbon

receptor (AhR) – whereas the other two are non-receptor-mediated – DNA damage (DNA_Damage) or cytotoxicity (Cytotoxic). More details of the study design and sample collection are available in the TGxSEQC publication (6, 7).

## 1.4 Gene annotation conventions

For the TCGA breast cancer data set, uniquely aligned read counts were assigned to annotated genes (Ensembl annotation) with overlapping genomic coordinates in the hg19 human reference genome (9). After analysis, lists of DEGREEs were curated in favor of Ensemble-annotated genes with officially recognized protein-coding transcripts by cross-referencing against the against the Entrez Gene database (10).

For the TGxSEQC rat liver data set, uniquely aligned read counts were assigned to gene transcripts (RefSeq annotation) with overlapping genomic coordinates in the rn6 rat reference genome (11). After analysis, lists of significantly expressed transcripts were curated in favor of protein-coding sequences (NM_ accession prefix) and cross-referenced against the Entrez Gene database (10) to extract the identity of the DEGREEs encoding transcripts with differential expression levels. Final lists of DEGREEs and their differential expression metrics equal the average among differentially expressed transcripts grouped under non-duplicate gene symbols.

## 1.5 Independent filtering

Expression levels of individual genes were calculated as the normalized rate of deduplicated and uniquely aligned reads per million (RPM) total reads. To perform independent filtering, we fit empirically observed RPM grand means of genes among parametric distribution functions of the exponential family with non-negative support. The main metrics used to discriminate among parsimoniously fit models were based on minimization of the −Loglikelihood function, and both the corrected Akaike and the Bayesian Information Criteria (AICc and BIC, respectively). When multiple distributions showed comparable best-fit metrics, a choice was made for subsequent analysis in favor of the distribution with the simplest algebraic manipulations to calculate each distribution’s sufficient statistics based on sequencing read output. Genes with average RPM across all replicates less than the location parameter of the fit distribution function were discarded from further analysis.

## 1.6 Gene resolution weights via generalized linear modeling

To determine a relative metric of resolvable read-based expression, we performed generalized linear modeling (GLM) of observed RPM grand means of genes via: a) the natural parameter $\boldsymbol{\theta}$ of the best-fit parametric distribution to gene grand means used for independent filtering; and b) its matching canonical link function $g(\mu)$ (12). Resolution weights assigned to genes equal the estimated cumulative hazard of each gene’s linearized *transformant* set based on the empirical cumulative distribution of two-way ANOVA significance scores (gene×group) for *linear predictor* values estimated through the *canonical link function*.

For GLM, consider the true value of a response $\boldsymbol{Y}$ is calculated from sets of sampling observations *y* whose random errors follow a probability density function from the exponential family with the general form

$$\boldsymbol{P_Y}(y;\theta,\phi) = exp\left\{\frac{\boldsymbol{B}(\boldsymbol{\theta})\cdot\boldsymbol{F}(y)-\boldsymbol{G}(\boldsymbol{\theta})}{\boldsymbol{A}(\phi)}+\boldsymbol{C}(y,\phi)\right\}$$

where $\boldsymbol{\theta}$ and $\phi$ are distribution and dispersion parameters, respectively; when the natural parameter $\boldsymbol{B}(\boldsymbol{\theta})$ and the sufficient statistic $\boldsymbol{F}(y)$ are both identity functions and the dispersion function $\boldsymbol{A}(\phi)$ is fixed such that

$$\boldsymbol{B}(\boldsymbol{\theta}) = \boldsymbol{\theta},$$

$$\boldsymbol{F}(y) = y, \text{ and}$$

$$\boldsymbol{A}(\phi) = \phi$$

the distribution parameter $\boldsymbol{\theta}$ is referred to as the *canonical parameter*.

Now, consider a function $\boldsymbol{\eta}(\boldsymbol{X})$ exists which consists of linear combinations of independent variables $\boldsymbol{X}$ with latent coefficients $\boldsymbol{\beta}$, i.e.

$$\boldsymbol{\eta}(\boldsymbol{X}) = \boldsymbol{X\beta}.$$

As long as $\boldsymbol{\eta}(\boldsymbol{X})$ can be expressed in terms of a well-defined one-to-one transformation of the expected value for the dependent variable $\boldsymbol{Y}$ such that

$$\boldsymbol{\eta}(\boldsymbol{X}) = g(\mathrm{E}[\boldsymbol{Y}]) = g(\mu)$$

then $\boldsymbol{\eta}(\boldsymbol{X})$ is an invertible *linear predictor* (or linearizing *transformant*) that ties $\boldsymbol{X}$ to the mean $\mu$ of response variable $\boldsymbol{Y}$ via a transformation of response means $g(\mu)$ known as the *link function*. The particular link function $g(\mu)$ that makes the linear predictor and the canonical parameter coincide such that

$$\boldsymbol{\theta} = \boldsymbol{\eta}(\boldsymbol{X}) = \boldsymbol{X\beta}$$

and

$$g^{-1}(\boldsymbol{\theta}) = \mu = g^{-1}(\boldsymbol{X\beta})$$

is referred to as the *canonical link function*, and operates as an *error-normalizing transformation* that maps independent variables $\boldsymbol{X}$ directly to observed $\boldsymbol{Y}$ responses through linear combinations of mean values of the canonical parameter $\boldsymbol{\theta}$ with a density function $\boldsymbol{P_Y}(y; \theta, \phi)$ from the exponential family.

## 1.7 Weighed ANOVA inferential testing

Statistical tests of differential gene expression were performed using a weighed two-way ANOVA model (gene×group blocks; N≥3 replicates per group) of $\log_2$-transformed fold changes (Log2FC) in RPM relative to either the gene-wise grand mean or the mean in a control group; an omnibus RPM value equal to the threshold parameter from the best-fit distribution used for independent filtering was added throughout to circumvent issues in the calculation of Log2FC with zero-valued RPM data. Gene-wise significance of Log2FC variation based on resolution-weighed ANOVA inferential tests were adjusted per Benjamini-Hochberg method for multiple comparisons (13).

## 1.8 Gene-wise effect size filtering

For effect size filtering, we considered a minimum practical effect size $\delta_{Log2FC}>\delta_{Effect}$, where the gene-wise effect size

$$\delta_{\mathrm{Log2FC}} = 0.3 \times \sigma_{\mathrm{SSR}}$$

given

$$\sigma_{\mathrm{SSR}} = \sqrt[2]{SSR_{\mathrm{Log2FC}}/(N-1)}$$

corresponds to 5% of the Log2FC *regression* error spread estimated at a $6\sigma_{\mathrm{SSR}}$ level with respect to a gene's fitted grand mean; likewise, the minimum practical effect size for a gene

$$\delta_{\mathrm{Effect}} = 0.3 \times \sigma_{\mathrm{SSE}}$$

given

$$\sigma_{\mathrm{SSE}} = \sqrt[2]{SSE_{\mathrm{Log2FC}}/(N-1)}$$

equals 5% of the estimated spread in *observed* error about mean Log2FC values within statistical gene×group blocks estimated at a $6\sigma_{Log2FC}$ population level.

### 1.9 Detection criteria for differentially expressed genes (DEGs)

Differentially expressed genes (DEGs) were identified as those showing statistically significantly weighed ANOVA scores (FDR adj. $p<0.05$). In the case of experimental designs with low replication levels (N=3 replicates per group) additional requisites to classify statistically significant genes based on resolution-weighed ANOVA as DEGs included a minimum gene-wise effect size $\delta_{Log2FC}>\delta_{Effect}$ and *post hoc* pairwise-significance (Student's t-test $p<0.05$) for Log2FC differences within genes between two or more statistical groups.

### 1.10 Signal-to-noise reproducibility threshold

Transcriptome-wide signal-to-noise (SNR) thresholds of practical reproducibility of differential expression were estimated based on the spread of individual Log2FC measurements around means of gene×group blocks after independent filtering, and are equal to the projected 95% tolerance interval of transcriptome-wide residuals at a 95% confidence (14-16); for qPCR validation, minimum reproducible SNR corresponds to the same estimate when performed only among DEGs.

## 2 Supplementary Results

### 2.1 EPIG-seq Simulated Data

To perform independent filtering, we fit empirically observed RPM averages from each of the 20,000 simulated pseudogenes across the entire 140-pseudoreplicate set to parametric distribution functions, and discriminated among parsimoniously fit models by minimization of the −Loglikelihood function and two of its related metrics, the corrected Akaike and the Bayesian Information Criteria (AICc and BIC, respectively). The three best-fit parametric functions with non-negative support corresponded, in that order, to the log-logistic, generalized gamma, and ordinary lognormal distributions; among

them, we chose the lognormal distribution for subsequent data analysis because it offers the simplest algebraic manipulations of the three and its sufficient statistics (location and scale parameters $\mu$ and $\sigma$, respectively) are also the easiest to calculate directly from the original read output - all these features needed to perform GLM.

Next, we carried out additional fits to threshold distributions, and found that the 3-parameter lognormal distribution, which is equivalent to the lognormal distribution shifted to a positive and non-zero minimum RPM value $\gamma$, was equally powerful in fitting gene-wise average RPM than the ordinary lognormal distribution; furthermore, $\mu$ and $\sigma$ were virtually equivalent between both ordinary and threshold lognormal fits (2-parameter lognormal $\mu_{2P}$=1.06±0.03, $\sigma_{2P}$=1.98±0.02; threshold 3-parameter lognormal $\mu_{3P}$=1.05±0.03, $\sigma_{3P}$=1.99±0.02 [predicted mean±95% prediction CI]). We reasoned the threshold $\gamma_{3P}=2.4\times10^{-3}$ (95% prediction CI: $0.9\times10^{-3}$ – $3.5\times10^{-3}$), which represents the lowest possible value of RPM with mathematical support that can be explained by the best-fit threshold distribution model, was the best candidate value to perform independent filtering against across pseudogenes with simulated reads. In the case of this simulated data set, independent filtering against the threshold parameter $\gamma_{3P}$ did not exclude any pseudogenes from subsequent analysis – meaning all listed pseudogenes were "detectable" assuming the estimated error model of simulated read counts was properly fit by a lognormal distribution. This outcome to independent filtering rarely occurs in the analysis of *bona fide* experimental data sets; yet, having no pseudogenes excluded prior to inferential testing is consistent with the simulated data being tailored to exhibit a long dynamic range of read coverage, encompassing all pseudogenes and all pseudoreplicates, and using an "engineered" error model. It also illustrates how an arbitrarily specified independent filter may exclude genes with useful information from differential analysis because of low read counts, even though their observed levels of sequencing representation may be within the limits of the dynamic range for some experiments and not others.

To proceed with GLM, it is necessary to restate the lognormal probability density function in the general form of the exponential family. In the particular case of the 3-parameter lognormal distribution, this is only possible for $\boldsymbol{B}(\boldsymbol{\theta})=\log(\boldsymbol{\theta})$ given $\boldsymbol{\theta}=x-\gamma_{3P}$, where $x$ is an observed RPM value for a gene in a replicate. However, since $\mu_{3P}/\gamma_{3P}\approx\mu_{2P}/\gamma_{3P}\sim O(3)$ and $(\langle\!\langle x\rangle\!\rangle_{\text{pseudogene}}-\gamma_{3P})>0$ throughout, it is possible to implement GLM with a 2-parameter lognormal distribution instead for algebraic simplicity, since $\log(\boldsymbol{\theta})=\log(x-\gamma_{3P})\approx\log(x)$ corresponds to a normal distribution with an identity canonical link function $g(\mu)=\boldsymbol{\theta}$; therefore, the transformant set $\boldsymbol{\eta}(\boldsymbol{X})$ for LSTNR analysis of the *in silico* simulated dataset is

$$\boldsymbol{\eta}(\boldsymbol{X})=\boldsymbol{X\beta}=\log(x).$$

With a suitable linear predictor function at hand, transformant $\log(x)$ values were calculated for all individual pseudoreplicates, across all pseudogenes and conditions, and tested by two-way ANOVA to estimate transformant significance scores for each pseudogene; we found transformant FDR $p<0.05$ in 9,492 of the 20,000 total pseudogenes, meaning 47.46% of all pseudogenes showed variability in read counts in one or more groups that was statistically discernible (***resolvable***) from that of all pseudoreplicates combined. Then, the empirical cumulative distribution function of continuous-valued transformant significance scores of pseudogenes was used to calculate pseudogene cumulative hazard rates and assign them to each respective pseudogene as resolution weights in differential expression multivariate testing.

To extract relative expression measurements, we determined a reference expression value in each pseudogene equal to the average $\log_2$(RPM) in the baseline group. The advantage of establishing a $\log_2$(RPM) reference is that sample means and variation from individual replicates can be estimated for all groups *including the baseline condition and prior to inferential testing of significance*. This information is critical to fashion *a priori* estimates of practical effect size – particularly when analyzing data from underpowered, biased, or low-replication experimental designs. For example, one could calculate expectable confidence intervals of signal dispersion based on observed $\log_2$(RPM) residuals from the means of pseudogenes within conditions, *regardless of statistical significance scores*, and define practical signal-to-noise threshold of reproducibility to validate against in qPCR confirmatory assays. For the EPIG-seq simulated data set, $\log_2(\text{RPM})^{95\%TI}=\pm 7.7$, meaning were these data derived from a "true" sample of biological specimens, one could project with up to 95% confidence, and based solely on the spread of residuals, that 95% of gene fold-change averages measured in repeat experiments may differ as much as ~200-fold from "true" population means.

To perform DEG discrimination we combined log-fold differences with each gene's resolution weights in a two-way multivariate ANOVA model. We reasoned that scaling relative expression measurements would also homogenize the scale of variation around the means of individual genes in each experimental statistical group. This was the assumption behind using a cumulative hazard function that scores non-linear variations in accrued reads per gene across the dynamic range of gene detection as a weight function; in doing so, the LSTNR method regularizes the estimated relative expression measurement errors of all replicates across all genes and allows for significance testing through ordinary multivariate ANOVA tests. When evaluating the distribution of Log2FC residuals we found, as postulated, that introducing resolution weights improved their range-scaled dispersion and homogeneity throughout. Finally, after multiple testing adjustment by the false-discovery rate approach (13), LSTNR's resolution-weighed two-way ANOVA approach detected a pool of 4,541 statistically significant pseudogenes (FDR adj. $p<0.05$).

We found that the pool of statistically significant pseudogenes contained all of the differentially expressed pseudogenes from the five simulated co-expression patterns; yet an added pool of 3,541 pseudogenes from the random noise "unpatterned" group were also captured. One could presume that the "unpatterned" pseudogenes with statistically significant expression levels detected through LSTNR, although not detrimental to detecting any of the "true" differential pseudogenes, could instead undermine the capacity to discriminate the "true" patterns of coordinated pseudogene expression, since excessive contributions of "noise" from unpatterned pseudogenes may compromise the agglomerative performance of traditional cluster analysis routines. To confirm the ability of LSTNR to replicate underlying patterns of expression accurately among detected pseudogenes, despite "unpatterned" pseudogenes accounting for most of the detected differential pseudogenes overall, we performed naïve hierarchical clustering (Ward's method). We successfully confirmed 100% discrimination between the 1,000 "true" and 3,541 "unpatterned" differential pseudogenes. Furthermore, we confirmed agreement in the assignment of "true" differential pseudogenes among five clades of expression trends to their simulated patterns of origin in 927 of the 1,000 patterned pseudogenes (contingency analysis Pearson's $p<0.0001$); of those 927 matching pseudogenes, 400 were classified into clades exactly matching their simulated counterparts (patterns B and D), meanwhile the remaining 527 pseudogenes belonged to simulated patterns consistently higher levels of expression in all groups v. the baseline (patterns A, C and D). Finally, we found clade-wise average expression levels detected by clustering analysis across groups matched the patterns of expression originally simulated *in silico*. We found the same results when discriminating the pool of

differential pseudogenes by clustering of their Pearson product-moment correlation matrix of Log2FC measurements.

# 3 Supplementary Discussion

## 3.1 qPCR and RNAseq: same problems, different indicators

Technical problems that can plague qPCR data analysis have equal counterparts in RNAseq bioinformatics because RNAseq relies on PCR-based amplification of a diverse library of DNA templates *en masse*. One particularly notorious hurdle pertains the amount of doubling needed to detect a signal: although PCR can generate billions of copies per initial template molecule after a few dozens of cycles, it also carries the risk of introducing base integration artifacts along the way which are propagated thereafter in the same exponential pace. In the case of qPCR, this implies rare templates will become indistinguishable from instrumental noise or trace contaminants the more amplification cycles are carried out to detect them, whether this is due to reaction artifacts or experimental variation. The issue is more complex in RNAseq: since different templates in the initial library are distinguished by how accurately their PCR-aggregated copies emulate the starting sequences, polymerase errors during base-pair integration can create multiple degenerate copies from the same original template, which accumulate exponentially, and resemble aggregates derived from different templates better as they accrue more PCR cycles.

Another technical problem regards accumulation of template copies past exponential doubling and into a geometric amplification phase (also referred to as overduplication). In overduplication, the transition from exponential to geometric doubling is not simultaneous between two samples when their initial template contents are different; this phenomenon can happen after free nucleotide pools in the reaction are exhausted or when the number of PCR copies from a given template becomes so dense that they impose stearic hindrances to DNA polymerase binding and processivity within a fixed reaction volume. In the context of qPCR assays, asynchronous amplification between two templates invalidates retrospective estimates of relative expression based on Ct values because the assumption of exponential amplification does not hold (17, 18). In RNAseq, overduplication leads to a library of unevenly amplified DNA fragments; in turn, the probability of capture onto a sequencing flow cell of overduplicated fragments is inflated with respect to the rest of the library, which not only biases the number of uniquely aligned reads assigned to genes expressed at different levels – it also worsens the dropout rate of raw collected reads during bioinformatics processing, when reads with duplicate sequences are thrown out to prevent double-counting of PCR copies from a single initial transcript in differential expression analyses, thereby undermining the return-on-investment of RNAseq runs based on the final output of useful reads uniquely aligned to a reference genome.

Molecular biologists are quite familiar with technical challenges in qPCR assays. In the context of targeted qPCR, rules-of-thumb for quality control include using positive and negative control templates, standard curves and limiting dilutions to establish detection and resolution limits of each assay. Similar metrics are not as clear-cut for RNAseq technologies; for example, decisions over what thresholds are appropriate to distinguish expressed genes from genes harboring artefactual reads are often left to the discretion of bioinformatics analysts in turn. Sometimes this is not a critical problem in terms of detecting DEGs from particular datasets under study; other times, however, it undercuts the degree at which findings from one dataset can be reproduced by the same group in separate experiments, by different analysts on the same dataset, or across research groups altogether.

### 3.2 Data-driven approach to independent filtering

The first step in the LSTNR method is to determine which genes are most relevant for differential expression analysis based on their sequencing output. This task involves fitting the empirical distribution of sequencing depths among genes to a non-negative parameterized distribution from the exponential family with two objectives in mind. The first objective is to identify genes sequenced at extremely low and poorly resolved depths, which inflate the magnitude and statistical significance of between-group gene expression differences, and discard them preemptively before performing inferential tests of differential expression; this is referred to as independent filtering. The second objective is to reconcile non-linear trends in the variation of aligned read counts across the dynamic range of gene coverage, and account for them in ranking genes and their expression differences; this is one approach to benchmarking instrumental resolution.

Any high-depth sequencing run will generate artefactual sequences at rates consistent with inaccuracies in hardware readout of reversible terminators or polymerase-driven nucleotide integration. Some of those reads will align by mere coincidence to gene coordinates in the reference genome. Consequently, the low read counts of such genes reflect the limits of instrumental resolution in sequencing. Statistically speaking, a low-count gene is indistinguishable from background if its number of aligned reads from a run is at the order of magnitude of expectable artefactual sequences that coincidentally align to the reference genome. Recognizing this notion also stresses how gene filtering can fail based using an arbitrary "read count cut-off". This can lead to reproducibility challenges even if the same cut-off is used across different experiments. On the one hand, genes with accrued read counts within the limits of the dynamic range in one experiment may be thrown out incorrectly in another – for example, if two samples from equal experimental groups are run separately, and produce different numbers of total read counts, the sequencing noise may not be the same. On the other hand, in what is commonly referred to as "oversequenced" or "saturated" specimens, background-level genes may be retained for statistical testing erroneously; this can occur in low-diversity libraries in which, once most sequencing fragments are bound, any remaining clears in the flow cell are occupied by trace and carry-over contaminants (19).

Independent filtering of genes, in common practice, is based on a convention of read count thresholds defined in a somewhat arbitrary fashion, which seem based on the performance of earlier sequencing platforms – e.g. genes with >10 cumulative RPM across all samples in a 2×3 experimental design. However, the incidence of artefactual reads grows with ensembled sequencing depth, and the number of genes retained for differential expression analysis after independent filtering often remains too high in RNAseq data sets that show one or many of the following characteristics: a) sequenced to saturation or multiplexed to high coverage levels, e.g. 100M+ reads per specimen; b) multiple groups under comparison, e.g. control and >2 alternate conditions; c) high replicative levels, e.g. clinical data with N>3 per condition; d) time course experiments with >2 sampling intervals. New and upcoming sequencing platforms have evolved to offer increasing sequencing depths per single run, which can enhance the granularity of differential expression measurements between experimental groups (often in benefit of statistical significance). Still, generating more reads increases the expectable numbers of artefactual aligned sequences, and with it the net read counts underresolved genes should be filtered against also goes up. This means a fixed independent filtering threshold grows inadequate as sequencing depth increases.

## References


1. Li J & Bushel PR (2016) EPIG-Seq: extracting patterns and identifying co-expressed genes from RNA-Seq data. *BMC genomics* 17:255.
2. Cancer Genome Atlas N (2012) Comprehensive molecular portraits of human breast tumours. *Nature* 490(7418):61-70.
3. Parker JS*, et al.* (2009) Supervised risk predictor of breast cancer based on intrinsic subtypes. *Journal of clinical oncology : official journal of the American Society of Clinical Oncology* 27(8):1160-1167.
4. Perou CM*, et al.* (2000) Molecular portraits of human breast tumours. *Nature* 406(6797):747-752.
5. Sorlie T*, et al.* (2001) Gene expression patterns of breast carcinomas distinguish tumor subclasses with clinical implications. *Proceedings of the National Academy of Sciences of the United States of America* 98(19):10869-10874.
6. Wang C*, et al.* (2014) The concordance between RNA-seq and microarray data depends on chemical treatment and transcript abundance. *Nature biotechnology* 32(9):926-932.
7. Gong B*, et al.* (2014) Transcriptomic profiling of rat liver samples in a comprehensive study design by RNA-Seq. *Sci Data* 1:140021.
8. Wheeler DL*, et al.* (2008) Database resources of the National Center for Biotechnology Information. *Nucleic acids research* 36(Database issue):D13-21.
9. Kent WJ*, et al.* (2002) The human genome browser at UCSC. *Genome research* 12(6):996-1006.
10. Maglott D, Ostell J, Pruitt KD, & Tatusova T (2007) Entrez Gene: gene-centered information at NCBI. *Nucleic acids research* 35(Database issue):D26-31.
11. O'Leary NA*, et al.* (2016) Reference sequence (RefSeq) database at NCBI: current status, taxonomic expansion, and functional annotation. *Nucleic acids research* 44(D1):D733-745.
12. Nelder JA & Wedderburn RW (1972) Generalized Linear Models. *J R Stat Soc Ser a-G* 135(3):370-+.
13. Benjamini Y & Hochberg Y (1995) Controlling the False Discovery Rate: A Practical and Powerful Approach to Multiple Testing. *Journal of the Royal Statistical Society. Series B (Methodological)* 57(1):289-300.
14. Hahn GJ & Meeker WQ (1991) *Statistical intervals : a guide for practitioners* (Wiley, New York) pp xvii, 392 p.
15. Odeh RE & Owen DB (1980) *Tables for normal tolerance limits, sampling plans, and screening* (M. Dekker).
16. Tamhane AC & Dunlop DD (2000) *Statistics and data analysis : from elementary to intermediate* (Prentice Hall, Upper Saddle River, NJ) pp xiv, 722 p.
17. Livak KJ & Schmittgen TD (2001) Analysis of relative gene expression data using real-time quantitative PCR and the 2(-Delta Delta C(T)) Method. *Methods* 25(4):402-408.

18. Pfaffl MW (2001) A new mathematical model for relative quantification in real-time RT-PCR. *Nucleic acids research* 29(9):e45.

19. Tarazona S, Garcia-Alcalde F, Dopazo J, Ferrer A, & Conesa A (2011) Differential expression in RNA-seq: a matter of depth. *Genome research* 21(12):2213-2223.
